# Exploration of the molecular mechanism of melatonin against polycystic ovary syndrome based on a network pharmacology approach and experimental validation

**DOI:** 10.3389/fendo.2025.1528518

**Published:** 2025-08-05

**Authors:** Ran Cheng, Ying Zhu, Sheng-kai Wang, Chun-Xiao Zong, Hong-Li Zhao, Qin Zhang

**Affiliations:** ^1^ Department of Traditional Chinese Medical Gynecology, Hangzhou Traditional Chinese Medicine (TCM) Hospital Affiliated to Zhejiang Chinese Medical University, Hangzhou, China; ^2^ Research Institute of Women’s Reproductive Health, Zhejiang Chinese Medical University, Hangzhou, China; ^3^ Zhejiang Key Laboratory of Precise Protection and Promotion of Fertility, Hangzhou, China; ^4^ School of Basic Medical Sciences, Zhejiang Chinese Medical University, Hangzhou, China; ^5^ Department of Pathology, Zhejiang Cancer Hospital, Hangzhou, China

**Keywords:** melatonin, polycystic ovary syndrome (PCOS), network pharmacology, circadian rhythm, hyperandrogenism

## Abstract

**Background:**

Among women of childbearing age, polycystic ovary syndrome (PCOS) is the predominant etiology of anovulatory infertility. Recent research has elucidated the role of melatonin as a medicinal agent in PCOS, especially hyperandrogenism. However, the precise mechanisms underlying its therapeutic efficacy remain largely unknown. This study integrated network pharmacology, molecular docking, molecular dynamics simulations, and laboratory confirmation to explore the pharmacological mechanisms of melatonin in PCOS.

**Methods:**

First, we conducted animal studies to evaluate the therapeutic efficacy of melatonin by administering it to circadian disruption-induced PCOS-like rats. Prospective medicinal targets of melatonin were acquired from databases such as DrugBank, Traditional Chinese Medicine Systems Pharmacology, PharmMapper, and SwissTarget Prediction. Targets related to PCOS were extracted from three databases: DisGeNET, GeneCards, and the National Center for Biotechnology Information gene. To visualize the relationships between proteins, a protein-protein interaction network was generated using the STRING database. Further investigation of these targets involved analyzing protein-protein interaction networks and conducting GO/KEGG enrichment analysis. Molecular docking techniques were employed to examine the interactions between melatonin and crucial targets. Molecular dynamics simulations were performed to confirm the stability of the association between the hub targets and the melatonin ligand. Finally, animal studies validated the effect of melatonin on the identified targets.

**Results:**

Animal experiments showed that melatonin ameliorated hyperandrogenism and ovarian dysfunction in constant darkness-induced PCOS-like rats. Network pharmacology analysis demonstrated that melatonin exhibited multiple modulatory effects on circadian rhythm, reproductive processes, metabolic processes, and oocyte maturation. Cytoscape network analysis revealed seven key targets, of which AR and CYP19A1 showed the highest affinity for melatonin by molecular docking. The stability of the AR/CYP19A1-melatonin complex was verified through computational simulations using molecular dynamics techniques. Furthermore, animal experiments have validated that melatonin can regulate key genes associated with hyperandrogenism, including AR and CYP19A1.

**Conclusion:**

Through network pharmacology, molecular docking, and experimental validation, this study reveals how melatonin may ameliorate PCOS and hyperandrogenism. Results suggest melatonin’s effects involve androgen excess mitigation, though further validation is needed. This work provides insight into melatonin’s actions in circadian-associated PCOS.

## Introduction

Globally, polycystic ovary syndrome (PCOS) affects 5–18% of women of childbearing age. This complex, chronic condition significantly impacts reproductive, metabolic, and emotional health throughout a woman’s life (1). A hallmark reproductive feature of PCOS is ovarian dysfunction, characterized by hyperandrogenism and irregular or absent ovulation (2). The origins of PCOS are multifactorial and not yet fully elucidated, encompassing genetic predisposition, exposure to environmental toxins, dietary factors, and nutritional status (3). Modern lifestyle changes and erratic sleep habits often lead to disrupted circadian rhythms (4). Critically, substantial evidence now links circadian rhythm disturbances directly to PCOS pathogenesis, highlighting circadian rhythm disruption as a significant environmental risk factor (5).

The molecular basis of circadian rhythms lies in transcriptional-translational feedback loops, primarily involving CLOCK/BMAL1 and PER/CRY proteins. The suprachiasmatic nucleus (SCN) in the hypothalamus acts as the central pacemaker, synchronizing peripheral clocks throughout the body in response to light-dark cycles (6, 7). This circadian system regulates numerous biological processes, including endocrine function, metabolism, and sleep (8, 9). Perturbations in core clock genes, such as Brain and Muscle ARNT-like protein-1 (BMAL1), have been implicated in PCOS pathogenesis, particularly hyperandrogenism (10). Mechanistically, BMAL1 influences the expression of aromatase (CYP19A1), the enzyme converting androgens to estrogens, within estrogen-producing cells (11, 12). Reduced BMAL1 expression correlates strongly with peripheral and ovarian hyperandrogenism (10, 13).

Melatonin, the primary hormone secreted by the pineal gland in response to darkness, is a crucial regulator of circadian rhythms (14). Its effects are mediated primarily through membrane-bound receptors, MTNR1A and MTNR1B (15). Notably, melatonin receptors are expressed within ovarian structures, suggesting a role in regulating sex steroid production during follicular development (16). Polymorphisms in both MTNR1A and MTNR1B genes are associated with PCOS, further strengthening the link between circadian abnormalities and the syndrome (17–19).

Clinical evidence supports that PCOS patients exhibit reduced serum melatonin levels (5) and notably lower follicular melatonin concentrations (5, 20). Importantly, melatonin supplementation significantly reduces testosterone levels in women with PCOS (21, 22). Preclinical studies corroborate this, demonstrating that melatonin improves reproductive and metabolic phenotypes in various PCOS animal models (e.g., Letrozole or DHEA-induced) (23, 24). Collectively, melatonin may prove to be a viable therapeutic option for PCOS management, pending further investigation. However, the precise molecular mechanisms underlying melatonin’s therapeutic efficacy in PCOS remain largely unknown.

Constant darkness exposure provides a robust experimental model of circadian disruption (25). Recent studies show it reliably induces both reproductive (hyperandrogenism, irregular cycles) and metabolic hallmarks of PCOS in rats (10, 26, 27). Crucially, the characteristic downregulation of core clock genes (e.g., Bmal1) observed in these rats mirrors findings in women with PCOS. This model, therefore, offers valuable insights into circadian dysrhythmia-induced PCOS phenotypes and potential therapeutic strategies. Consequently, this study aimed to identify melatonin’s potential targets and elucidate its therapeutic processes in PCOS. We employed an integrated strategy combining network pharmacology, molecular docking, molecular dynamics simulations, and experimental validation in a constant darkness-induced PCOS-like rat model to explore the plausible mechanisms of melatonin action, providing foundational data for future preclinical research.

## Methods

### Preparing for constant darkness-induced PCOS-like rats

Eighteen female Sprague-Dawley (SD) rats of 5-week-old (Charles River Laboratory Animal Technology, Beijing, China) were housed in Animal Centre of Zhejiang Chinese Medical University (Hangzhou, Zhejiang, China), accredited by Association for Assessment and Accreditation of Laboratory Animal Care (AAALAC). The experimental animals were maintained in a specific-pathogen-free (SPF) grade conditions with temperatures ranging from 22°C to 24°C and humidity ranging from 40°C to 65°C. The rats had unrestricted access to regular feed and drinking water in their enclosures at the Laboratory Animal Research Center. The Animal Ethics Committee of Zhejiang Chinese Medical University authorized all experimental procedures (Approval No. 20220815-11).

**Figure 1:**
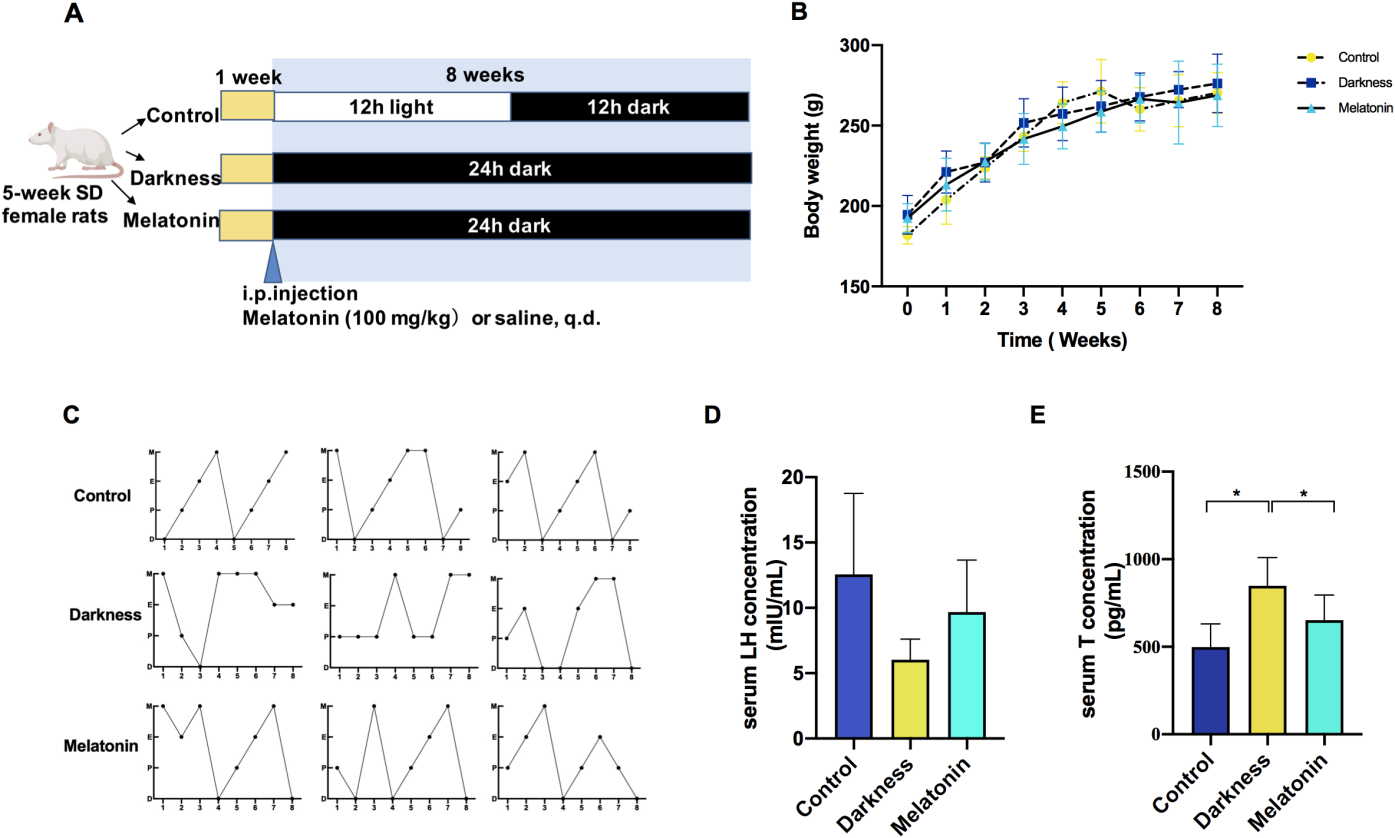
Melatonin treatment ameliorates reproductive hallmarks of PCOS in rats exposed to constant darkness. **(A)** Timeline of an experiment into darkness-induced PCOS and melatonin treatment. **(B)** Body weight changes, **(C)** Representative estrous cycles. M represents for metestrus; D represents for diestrus; P represents for proestrus; E represents for estrus, **(D, E)** Serum concentrations of LH and testosterone by ELISA. Statistical analysis **(D, E)** was performed with one-way ANOVA followed by Newman–Keuls multiple comparison test. N=6 per group. Data are expressed as mean± SD. *P < 0.05.

After adaptive feeding for 1 week, the rats were equally and randomly assigned three groups (by Ying Zhu): control, darkness, and melatonin group (*n*=6). **Figure 1A** illustrates the experimental design of the animal study. The control group was kept under a normal 12-hour light/dark (L/D) alternating exposure (illumination beginning at 7:30 AM, Zeitgeber Time 0, ZT0). Both the darkness and melatonin group were housed in constant darkness for 8 weeks. In the melatonin group, rats were intraperitoneally administered melatonin daily at ZT11, with a dosage of 10 mg/kg/day, which was widely preferred in rodent models (10, 28). The control and darkness group were intraperitoneally administered with saline of equal volume. Whole rat body weight was measured weekly throughout the 8-week period. Rats were sacrificed under the effects of 5% isoflurane anesthesia during the diestrus II stage for tissue sample collection.

### Vaginal cytology analysis

For eight consecutive days during rat model development, vaginal smears were examined to determine the estrous cycle stages (by Ran Cheng and Ying Zhu). The procedure involved collecting vaginal secretions from each rat between 9:00 and 10:00 a.m. by rinsing the vagina with a saline solution. Collected secretions were then placed on a glass slide for microscopic analysis. The estrous cycle stages were classified into four categories: metestrus (diestrus I), characterized by a small number of cornified cells and numerous leukocytes; diestrus (diestrus II), primarily composed of leukocytes; proestrus, distinguished by a high concentration of nucleated epithelial cells and estrus, dominated by large, flat, irregularly shaped a nucleated cornified epithelial cells.

### PBMC isolation

Peripheral blood mononuclear cells (PBMCs) were obtained from whole blood by standard Ficoll-Hypaque density centrifugation using rat peripheral blood lymphocyte isolation fluid (#KGA3103, KeyGEN Biotechnology) in accordance with the producer’s instructions (by Ran Cheng and Ying Zhu). The mononuclear cells underwent three washing cycles using PBS before being preserved at -80°C for subsequent extraction of total RNA.

### Hematoxylin and eosin staining

Rat ovarian tissues were harvested and fixed with 4% paraformaldehyde prior to paraffin embedding (by Sheng-kai Wang). Sections of tissue, 5 μm in thickness, were prepared and subsequently subjected to dewaxing and rehydration using a graded series of ethanol solutions. Tissue samples were colored with hematoxylin for 5 min, and differentiated with hydrochloric acid for 30 s. Finally, the specimens were stained with eosin for 2 minutes and mounted on slides for microscopic examination.

### ELISA

The concentrations of LH and testosterone in rat sera were detected using the Rat LH ELISA Kit (#ELK2367, ELK Biotechnology) and Testosterone ELISA Kit (#ELK1332, ELK Biotechnology), respectively (by Chun-xiao Zong). The protocols were performed in accordance with the guidelines.

### Determination of quantitative real‐time PCR

Total RNA from PBMCs and rat ovary tissues was isolated with TRIzol reagent (Invitrogen) and then reverse-transcribed into cDNA (#RR036A, Takara Bio, Dalian, China). RT-qPCR (#RR820A, Takara Bio, Dalian, China) was employed to measure the mRNA expression of target genes (by Ying Zhu). The ΔΔCt method was subsequently utilized for data analysis. The target mRNA level was calculated as the ratio of the target gene to GAPDH. **Table 1** tabulates the primer sequences used for the target genes.

**Table 1:** Primer pairs used for quantitative PCR analysis.

Gene	Primer forward (5′ to 3′)	Primer reverse (5′ to 3′)
AR	AAAAGAGCTGCGGAAGGGAA	TTTCCGGAGACGACACGATG
CYP19A1	ACTCATCATCAGCAAGTCCTCG	ACAGGCTCGGGTTGTTGTTA
MTNR1A	AAGCTCAGGAACGCAGGGAATA	AAGTATAGACGTCAGCGCCAA
MTNR1B	CTGTGGACTTCGTGGGGAAC	CACCACAAATAAATTACCTGCGT
BMAL1	GGCTGTTCAGCACATGAAAAC	GCTGCCCTGAGAATTAGGTGTT
CLOCK	CTTCCTGGTAACGCGAGAAAG	GTCGAATCTCACTAGCATCTGAC
PER2	CAGGTTGAGGGCATTACCTCC	AGGCGTCCTTCTTACAGTGAA
GAPDH	ACCACAGTCCATGCCATCAC	TCCACCACCCTGTTGCTGTA

### Identification of potential medicinal targets of melatonin

To obtain the spatial configuration and Simplified Molecular-Input Line-Entry Specification (SMILES) notation of melatonin, a search was conducted in the PubChem database (https://pubchem.ncbi.nlm.nih.gov/) (29). Subsequently, the 3D structure was input into the PharmMapper-server for potential target prediction, whereas SMILES was submitted to SwissTargetPrediction (http://www.swisstargetprediction.ch/) (30, 31). Furthermore, the DrugBank and Traditional Chinese Medicine Systems Pharmacology (TCMSP) (https://tcmspw.com/tcmsp.php) were utilized to recognized melatonin targets (32, 33). The retrieved target names were verified against authorized names in the UniProt database (https://www.uniprot.org/), and duplicate entries were eliminated. Cytoscape 3.8.2 was employed to visualize the results (by Ran Cheng and Chun-xiao Zong).

### Identification of PCOS-associated targets

To identify targets associated with PCOS, researchers (by Ran Cheng and Sheng-kai Wang) have utilized three gene databases: GeneCards, DisGeNET, and NCBI (34, 35). The search terms “Polycystic ovary syndrome” and “PCOS” were employed. To ensure result reliability, GeneCards had a minimum relevance score of minimum 0.7, and a gene-disease score of 0.1 or higher, was applied. The STRING database (version 11.5) was used to predict the network of interactions between proteins (PPI) for the given gene sets with a high-confidence interaction score (0.900). Cytoscape software 3.8.2 was then used to visualize the network construction.

### Protein-protein interaction network construction and discovery of hub genes

To identify potential medicinal targets, researchers (by Ran Cheng and Sheng-kai Wang) intersected melatonin and PCOS targets using the Venn Diagram tool. The PPI network of the gene lists was predicted using the STRING database with a moderate confidence interaction score (0.400) (36). The Cytoscape plugin “CytoHubba” was employed to identify hub genes using three topological analysis methods: Maximal Clique Centrality (MCC), Maximum Neighborhood Component Centrality (DMNC), and Neighborhood Component Centrality (MNC) (37).

### Gene Ontology, Kyoto Encyclopedia of Genes and Genomes enrichment analysis

DAVID (Database for Annotation, Visualization, and Integrated Discovery) equips researchers with an extensive array of functional characterization instruments, enabling them to uncover the biological relevance of substantial gene collections (38). We employed DAVID tools to conduct GO and KEGG pathway enrichment analyses to elucidate the functions of the potential medicinal targets as well as signaling pathways (by Ran Cheng and Sheng-kai Wang). The results of these analyses were visualized using a freely available online bioinformatics platform (https://www.bioinformatics.com.cn/).

### Molecular docking

The Protein Data Bank (39) supplied the 3D crystal structures of the target proteins in the PDB format. The PyMOL software (40)was used to eliminate water molecules and excess ligands. Subsequently, AutoDock tools (1.5.6) were utilized to incorporate protons and generate the PDBQT format of the proteins (41).

The 2D SDF structure of melatonin was obtained from PubChem (42). Energy minimization was performed using Chem 3D software (21.0.0) and the mol2 format was created (43). AutoDock tools (version 1.5.6) were then used to create the PDBQT format of melatonin.

Molecular docking with AutoDock Vina (40) was employed to assess melatonin binding and the hub pivot targets. Prior to molecular docking, proteins were represented as secondary structures. AutoDock Vina computed the binding affinity, while PyMol 2.5.4 (open-source version) was displayed to visually render the docking results (by Ran Cheng).

### Molecular dynamic simulation

The configurations exhibiting the two leading scores derived through molecular docking were subsequently subjected to molecular dynamics (MD) simulations utilizing Gromacs 2022.3 software (44, 45). The preparation of small molecules involved pretreatment with AmberTools22 and hydrogenation using Gaussian 16 W to determine the response potential. The computed potential data were incorporated into the MD system’s topology file. Throughout the simulation, the conditions were kept constant at 300 K and 1 bar pressure. The simulation employed the amber99sb ildn force field, and Tip3p was chosen as the water model for solvation. To achieve a neutral total charge in the simulated system, an appropriate amount of Na+ ions was introduced. The energy of the simulation system was minimized through a two-step process: first, the steepest descent technique was employed, followed by equilibration procedures. These included 100000 steps each of NVT (constant volume and temperature) and NPT (constant pressure and temperature) equilibration, utilizing a coupling constant of 0.1 ps over a 100ps time period. After equilibration, a free molecular dynamics simulation was performed, comprising five million steps with a 2fs step size, amounting to 100ns. A post-simulation assessment was conducted using the built-in tools of the software. This analysis encompassed the calculations of RMSD, RMSF, and rotational radius of the protein for each amino acid trajectory, along with free energy (MMGBSA) computations and generation of free energy topographic maps, among other relevant data (by Ran Cheng). These structural predictions provide a theoretical basis for the potential mechanism of action, but they need to be comprehensively analyzed in conjunction with the expression changes at the transcriptional level.

### Statistical analysis

In the Network Pharmacology section, topological analysis was performed using Cytoscape 3.8.2. The DAVID tools were applied for GO and KEGG enrichment analyses. AutoDock tools (1.5.6) were utilized to construct molecular docking simulations with PyMol 2.5.4, which were constructed using Gromacs 2022.3 software. Data evaluation was performed using GraphPad Prism 8 and SPSS version 25. As for the enrichment analysis, statistical significance was determined by a *P* -value < 0.05. In the section of experimental validation, results are expressed as mean ± SD. Each experiment was performed three times on an average. Data were first analyzed using the Kolmogorov–Smirnov test to verify the standard distribution. In cases where the data exhibited a normal distribution, statistical analyses were performed using either a paired Student’s t-test or one-way ANOVA, with subsequent Newman-Keuls *post hoc* testing. As for non-normally distributed data, the Kruskal–Wallis test followed by Dunn’s *post-hoc* test was applied. The threshold for statistical significance was established at *P* -value < 0.05(two-sided). This is denoted by *P < 0.05, **P < 0.01, and ***P < 0.001.

## Results

### Melatonin ameliorate hyperandrogenism and ovarian dysfunction in constant darkness-induced PCOS-like rats

**Figure 2:**
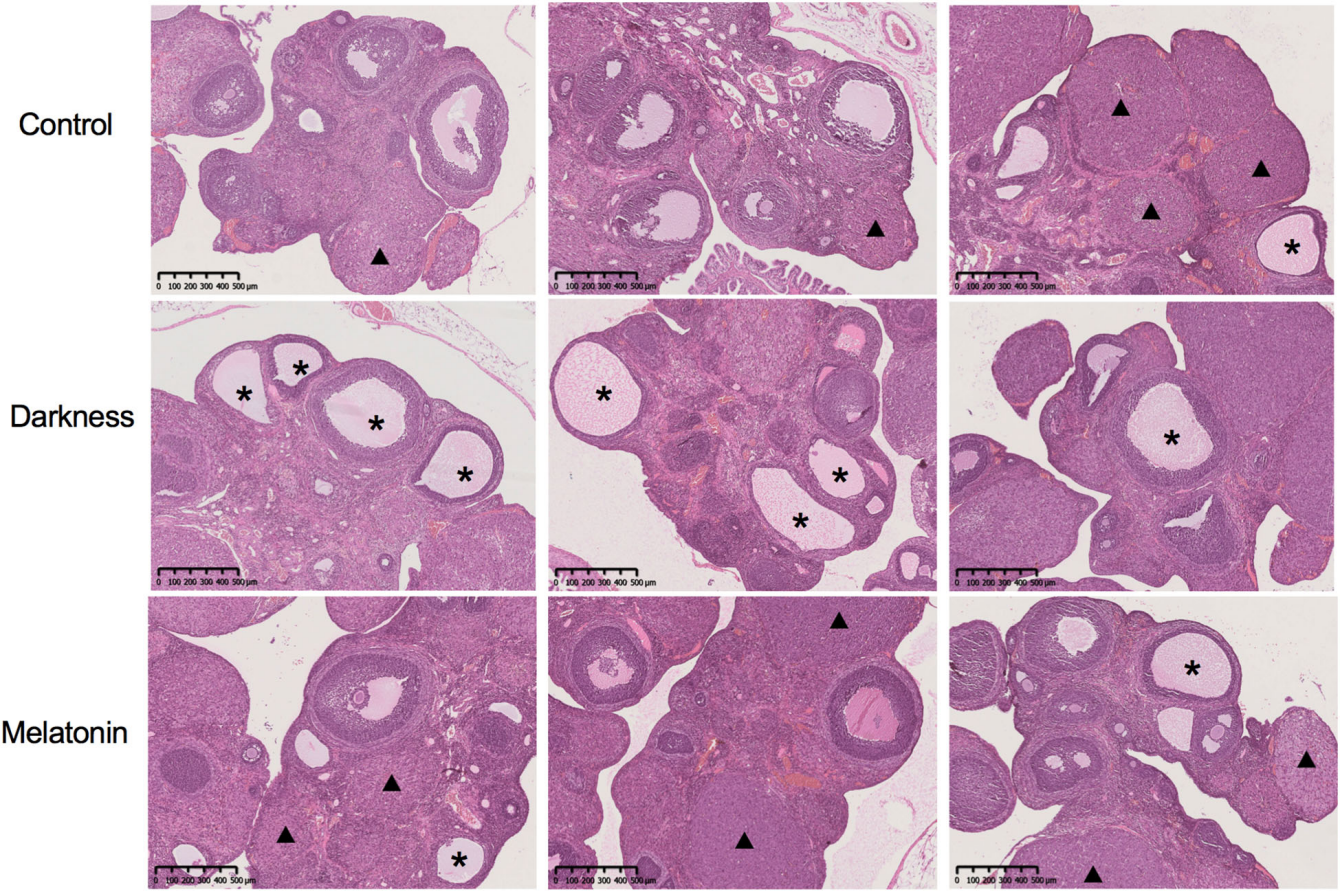
The ovarian and follicular morphologies of rats were assessed by hemoxylin and eosin staining. Asterisk stands for cystic follicles, triangle stands for the corpus luteum. Scale bar = 500 μm. N = 6 *per* group.

To examine how melatonin affects PCOS-like characteristics induced by circadian rhythm disruption, we administered melatonin injections to female Sprague–Dawley rats over an 8-week period while housing them in continuous darkness (**Figure 1A**). Rat-body-weights in the different groups were identical (**Figure 1B**). In the PCOS-like rat model subjected to constant darkness, we noted several characteristic features. These included irregular estrous cycles (**Figure 1C**), an increase in the quantity of abnormally large follicles, downregulation of ovarian corpora lutea (**Figure 2**), and elevated serum testosterone levels (**Figure 1E**), which were consistent with previous studies (10, 26, 27). But the serum LH level remained unchanged (**Figure 1D**). The melatonin administration improved the disrupted estrus cycles and decreased the number of antral follicles in darkness rats. Besides, melatonin treatment also significantly alleviated the high serum testosterone level (**Figures 1E**, **2**).

### Melatonin targets network

A comprehensive set of 233 melatonin targets was compiled from PharmMapper (119 targets), SwissTargetPrediction (100 targets), DrugBank (17 targets), and TCMSP (7 targets). Among them, 10 duplicated targets were removed and subsequently illustrated using Cytoscape (version 3.8.2) (**Supplementary Figure S1**).

### PPI network of PCOS targets

Based on DisGeNET (262 targets), GeneCards (436 targets), and NCBI Gene (457 targets), 803 PCOS targets were identified. Utilizing Cytoscape’s analysis network feature in the string interaction network, we identified 103 significant targets for PCOS. These targets exhibited above-average values for a degree of centrality of 14.00000, betweenness centrality of 0.00348, and closeness centrality of 0.33856 (**Supplementary Figure S2**).

### PPI network construction

**Figure 3:**
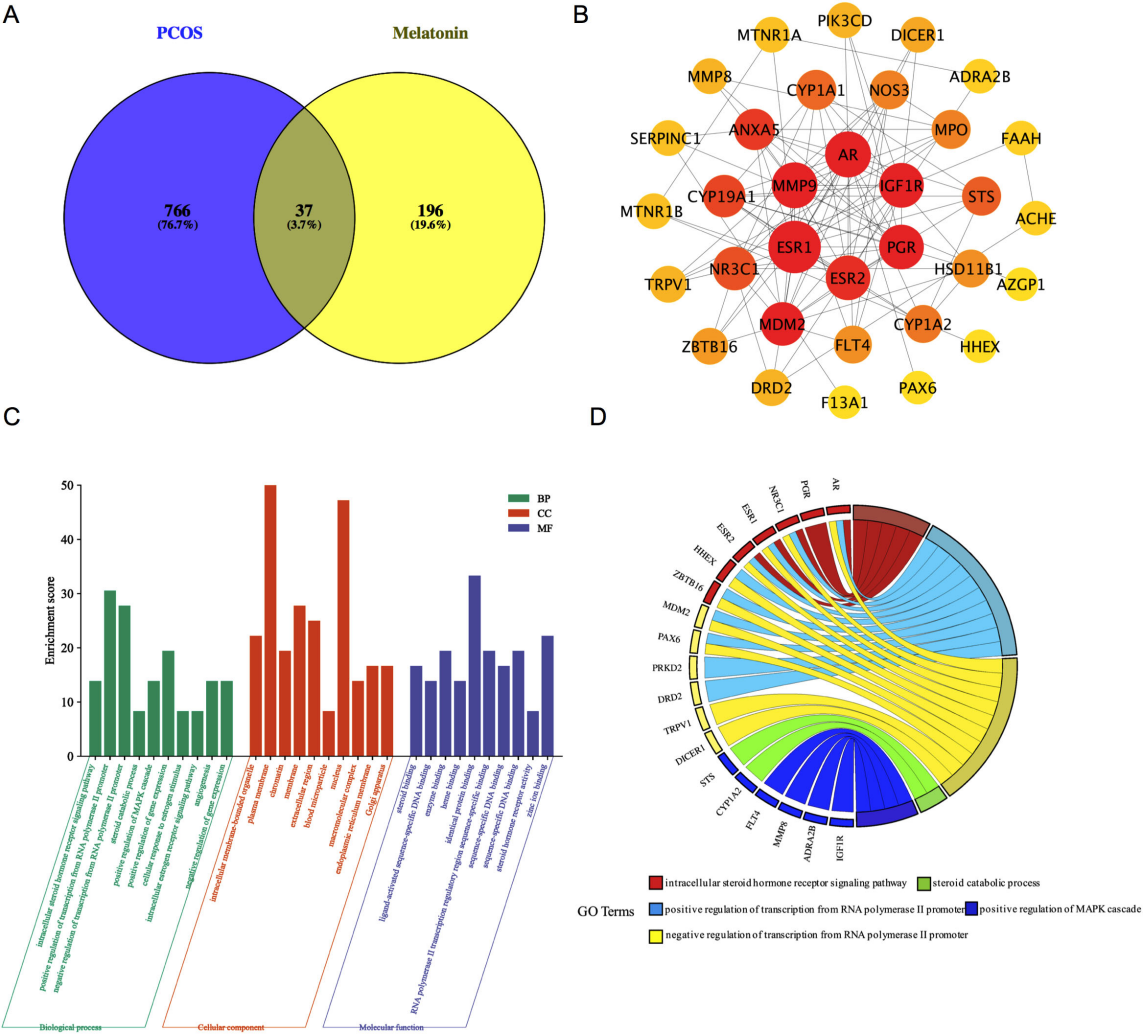
The Venn diagram and PPI network of melatonin predictive targets for PCOS. **(A)** The common targets of melatonin and PCOS. **(B)** The PPI network of 37 common target genes with a medium confidence interaction score of 0.400. PPI, protein-protein interaction; PCOS, polycystic ovary syndrome **(C)** The top 10 GO terms in the enrichment analysis in BP, CC, and MF. **(D)** The top five enriched biological processes. BP, biological processes; CC, cellular components; MF, molecular function; GO, Gene Ontology.

By applying the Venn Diagram tool, researchers (Ran Cheng and Sheng-kai Wang) identified 37 potential targets related to melatonin and PCOS (**Figure 3A**). Subsequently, we utilized the STRING database to generate a 37-co-targets’ PPI-network (**Figure 3B**). The MCC, DMNC, and MNC scores were calculated by “CytoHubba” plugin, and the intersection of hub targets [AR, CYP19A1, PGR, IGF1R, MDM2, NR3C1, and CYP1A1] was obtained.

### GO analysis

DAVID tools were employed to analyze the 37 common targets, with the top 10 Gene Ontology (GO) terms selected based on P-value and count frequency. **Figure 3C** displays the GO results, which were represented through a platform (free online: https://www.bioinformatics.com.cn/). The five most prominent biological processes (BP) were recognized in line with their count frequency and P-value, as shown in **Figure 3D**. These key BPs were found to be associated with melatonin treatment for PCOS: the intracellular steroid hormone receptor Signaling pathway, the positive regulation of the MAPK cascade, the steroid catabolic process, and the positive and negative regulation of transcription from the RNA polymerase II promoter. Importantly, four of the seven hub genes (AR, PGR, NR3C1, and MDM2) were enriched among the five leading BPs.

### KEGG pathway enrichment analysis

**Figure 4:**
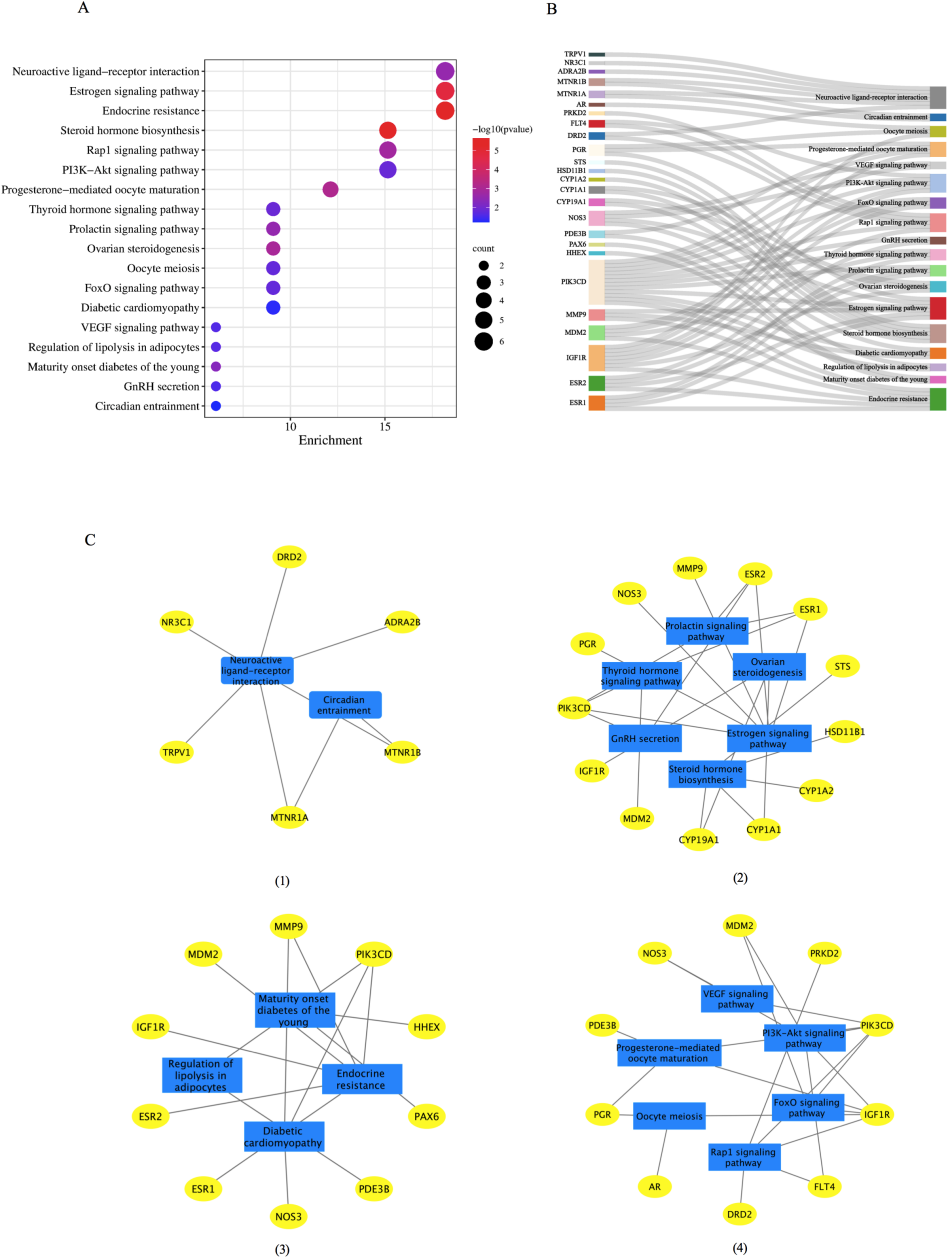
The KEGG functional analysis of the 37 common target genes. **(A)** The 18 significant pathways. The bubble sizes are indicated from large to small in descending order of the count of the potential target genes enriched in the pathways. The bubble colors are shown from red to blue in descending order of −log10(P value). **(B)** Target-pathways network. **(C)** Module analysis of the 18 KEGG pathways. Four modules were obtained according to the function of KEGG: (I) module 1, circadian rhythm; (II) module 2: reproductive process, (III) module 3, metabolic process; (IV) module 4, oocyte maturation. GnRH, gonadotrophin-releasing hormone; KEGG, Kyoto Encyclopedia of Genes and Genomes.

DAVID tools were employed to conduct a KEGG pathway enrichment analysis on the 37 therapeutic targets, identifying 18 pathways with significant associations, as depicted in **Figure 4A**. The targets enriched within individual pathways are shown in **Figure 4B**. These 18 pathways were organized into four functional modules, as shown in **Figure 4C**. Module 1 incorporated two pathways: circadian entrainment (hsa04713) and neuroactive ligand-receptor interaction (hsa04080). Module 2 encompasses six pathways: steroid hormone biosynthesis (hsa00140), ovarian steroidogenesis (hsa04913), prolactin signaling (hsa04917), estrogen signaling (hsa04915), GnRH secretion (hsa04929), and thyroid hormone signaling (hsa04919). Module 3 contained four pathways: diabetic cardiomyopathy (hsa05415), regulation of lipolysis in adipocytes (hsa04923), maturity-onset diabetes of the young (hsa04950), and endocrine resistance (hsa01522). Module 4 was composed of six pathways: the PI3K-Akt Signaling pathway (hsa04151), VEGF Signaling pathway (hsa04370), Rap1 Signaling pathway (hsa04015), FOXO Signaling pathway (hsa04068), oocyte meiosis (hsa04114), and progesterone-mediated oocyte maturation (hsa04914).

### Molecular docking

**Figure 5:**
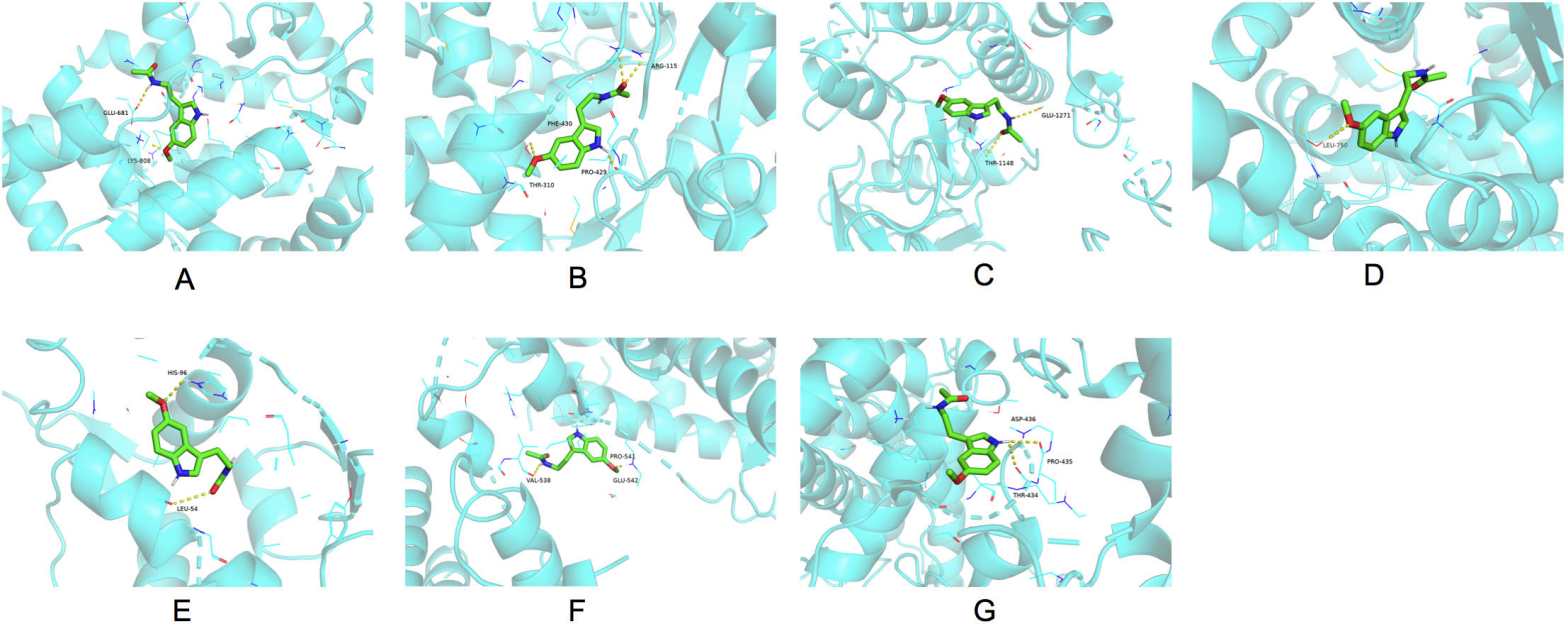
Molecular docking results of melatonin with the seven hub target proteins ranked by the MCC, DMNC and MNC algorithm of the “CytoHubba” plugin using the Cytoscape network analysis. The binding sites of melatonin with AR **(A)**, CYP19A1 **(B)**, PGR **(C)**, IGF1R **(D)**, MDM2 **(E)**, NR3C1 **(F)**, CYP1A1 **(G)**. MCC, Maximal Clique Centrality; DMNC, Maximum Neighborhood Component Centrality; MNC: Neighborhood Component Centrality; AR, androgen receptor; CYP19A1, cytochrome P450 family 19 subfamily A member 1; PGR, progesterone receptor; IGF1R, insulin-like growth factor I receptor; NR3C1, nuclear receptor subfamily 3 group C member 1; CYP1A1, cytochrome P450 family 1 subfamily A member 1.

Seven hub genes sorted by MCC, DMNC, and MCN were identified for molecular docking analysis with melatonin (**Figure 5**). **Table 2** outlined the specifications of the molecular docking parameters. A stronger binding ability was indicated by a lower docking affinity. The binding strength between melatonin and its target molecules was substantial, with affinity values ranging from −7.5 to −5.4 kcal/mol. Melatonin may effectively treat PCOS by modulating the activities of these significant targets.

**Table 2:** Details of molecular docking parameters.

Targets	PDB ID^1^	Box_center (x, y, z)	Box_size (x, y, z)	Affinity (kcal/mol)
AR	2PIU	10.408, 7.95, 11.619	62.78, 37.668, 50, 224	−7.5
CYP19A1	5JKV	80.84, 55.165, 55.111	66.85, 66.85, 66.85	−6.7
PGR	2OVH	31.62, 10.242, 27.79	50.906, 65.45, 63.372	−6.5
IGF1R	3LW0	1.475, −33.283, 47.626	126, 126, 126	−6.4
MDM2	5OAI	59.098, 6.143, 14.412	32.25, 32.25, 33	−6.1
NR3C1	3K22	−19.192, 37.482, 0.034	74.55, 74.55, 74.55	−6.0
CYP1A1	6UDL	19.399, −17.272, −24.009	126, 126, 126	−5.4

^1^PDB: Protein Data Bank.

### MD simulation

**Figure 6:**
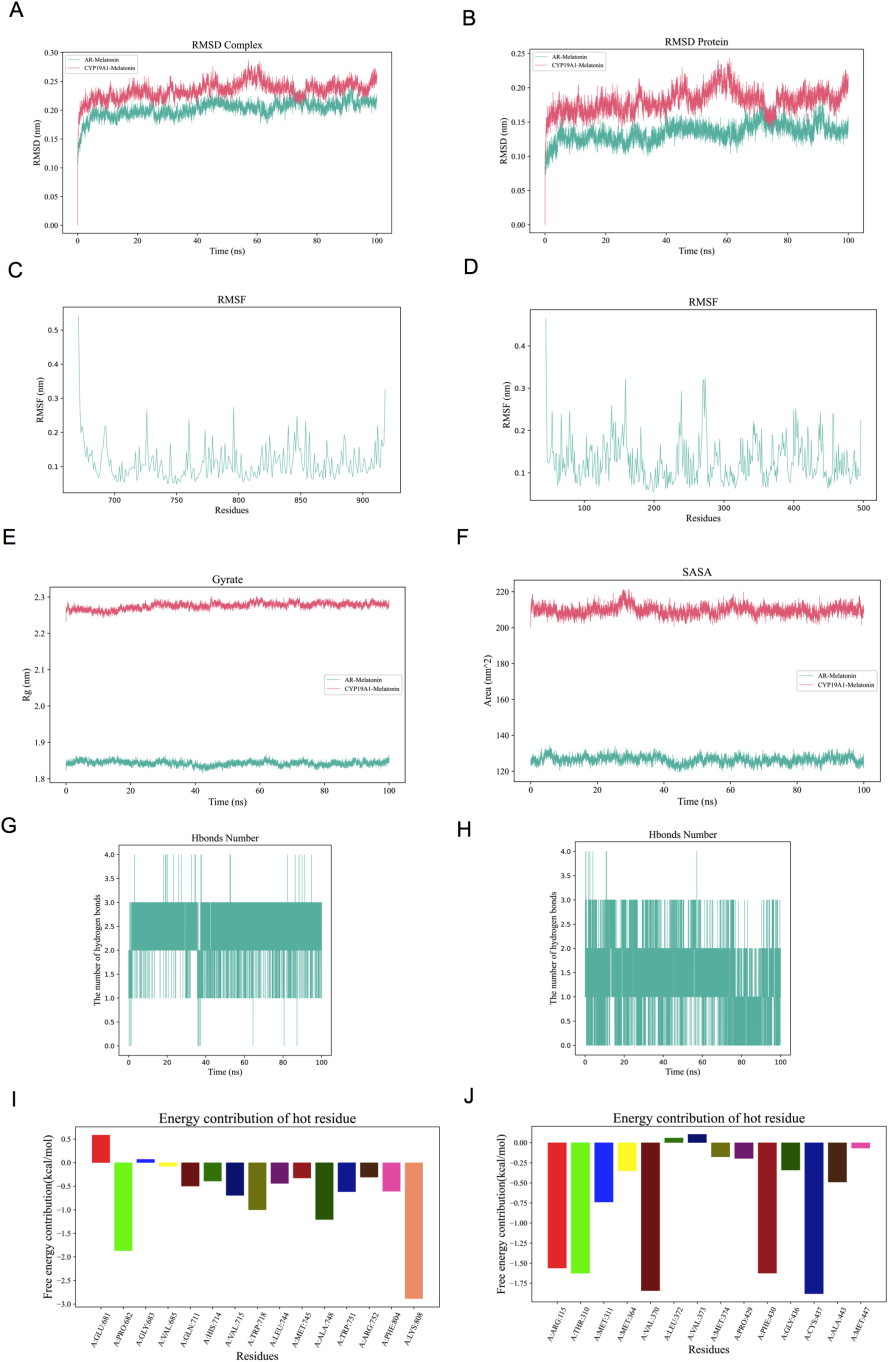
The molecular dynamics’ simulation of AR/CYP19A1–melatonin complex. **(A)** RMSD curve of the AR/CYP19A1–melatonin complex; **(B)** RMSD curve of melatonin; **(C)** RMSF curve of AR; **(D)** RMSF curve of CYP19A1; **(E)** Rg curve of the AR/CYP19A1–melatonin complex; **(F)** SASA curve of the AR/CYP19A1–melatonin complex; **(G)** Number of hydrogen bonds in the AR–melatonin complex; **(H)** Number of hydrogen bonds in the CYP19A1–melatonin complex; **(I)**Amino acid decomposition of the AR–melatonin complex; **(J)** Amino acid decomposition of the CYP19A1–melatonin complex.

MD simulations were performed to verify the molecular docking simulation results for AR and CYP19A1. The RMSD curves for the AR/CYP19A1-melatonin complexes showed equilibrium after 60 ns, with average RMSD values of 0.2 nm and 0.23 nm (**Figure 6A**). Evaluation of the molecular stability using RMSD measurements in relation to the starting conformation (**Figure 6B**) showed that melatonin exhibited the smallest positional shifts when complexed with AR. The Rg values for the two complexes-maintained equilibrium with means of 1.85 nm and 2.25 nm (**Figure 6E**). The average SASA values remained consistent at 128 nm² and 210 nm² (**Figure 6F**). Throughout the 100 ns simulation, the count of hydrogen-bonding interactions for the AR/CYP19A1-melatonin complexes ranged from to 1–4 and 1-3, respectively (**Figures 6G, H**). The RMSF analysis revealed crucial flexible areas within the AR and CYP19A1 proteins, particularly in the vicinity of specific amino acid residues 200–40 for AR-melatonin; 790–800 for CYP19A1-melatonin (**Figures 6C, D, I, J**). The complex’s Gibbs free energy profiles exhibited clear minimum energy basins (**Figures 6A, B, E**), accompanied by hydrogen bond counts of 3 and 2, respectively (**Figures 6B–D, G–F**). **Table 3** displayed the binding free energy of the complexes formed between AR/CYP19A1 and melatonin. The MD simulation generated movie-like files depicting the trajectories (**Supplementary Movie S1**. AR and **Supplementary Movie S2**. CYP19A1).

**Table 3:** Analysis of binding free energy of the AR/CYP19A1–melatonin complex (kcal/mol).

Item	AR-melatonin	CYP19A1-melatonin
Δ_VDWAALS_	-31.86 ± 0.52	-28.90 ± 0.48
ΔE_elec_	-21.38 ± 1.58	-32.07 ± 0.02
ΔE_GB_	28.24 ± 1.57	37.31 ± 0.60
ΔE_surf_	-4.27 ± 0.01	-4.22 ± 0.00
ΔG_gas_	-53.24 ± 1.66	-60.97 ± 0.48
ΔG_solvation_	23.97 ± 1.57	33.10 ± 0.60
ΔTotal	-29.27 ± 2.28	-27.87 ± 0.77

**Figure 7:**
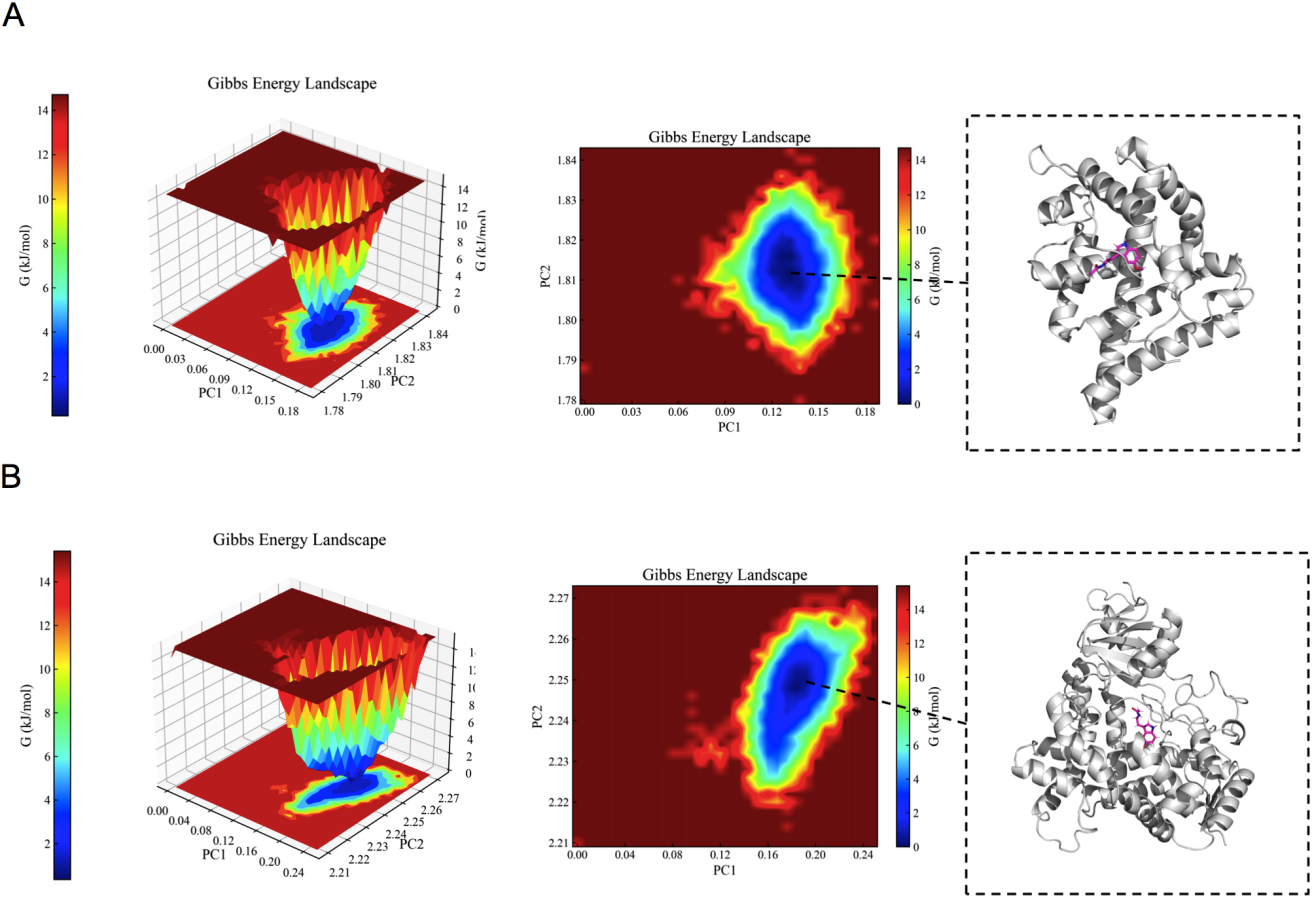
The results of the Gibbs free energy analysis. **(A)** 3D and 2D contour plots of the AR–melatonin complex. **(B)** 3D and 2D contour plots of the CYP19A1–melatonin complex.

As illustrated in **Figure 7**, the minimum value of the free energy is indicated by the blue region, while the metastable conformation is represented by the cyan and green regions. A higher prevalence of blue coloration was observed for AR/CYP19A1-melatonin complexes, suggesting favorable binding energetics and conformational stability within the simulation timeframe. Molecular dynamics (MD) simulations thus indicated the potential for stable physical interaction between melatonin and the identified target proteins. While this computational finding supports the plausibility of melatonin binding to these targets, it is important to note that MD simulations primarily assess structural dynamics and binding stability, not functional consequences. The potential biological significance of these interactions, such as modulation of enzymatic activity or transcriptional regulation relevant to hyperandrogenism, remains hypothetical and requires empirical validation through functional assays.

### Effect of melatonin on the mRNA expression of peripheral circadian genes in PBMCs

**Figure 8:**
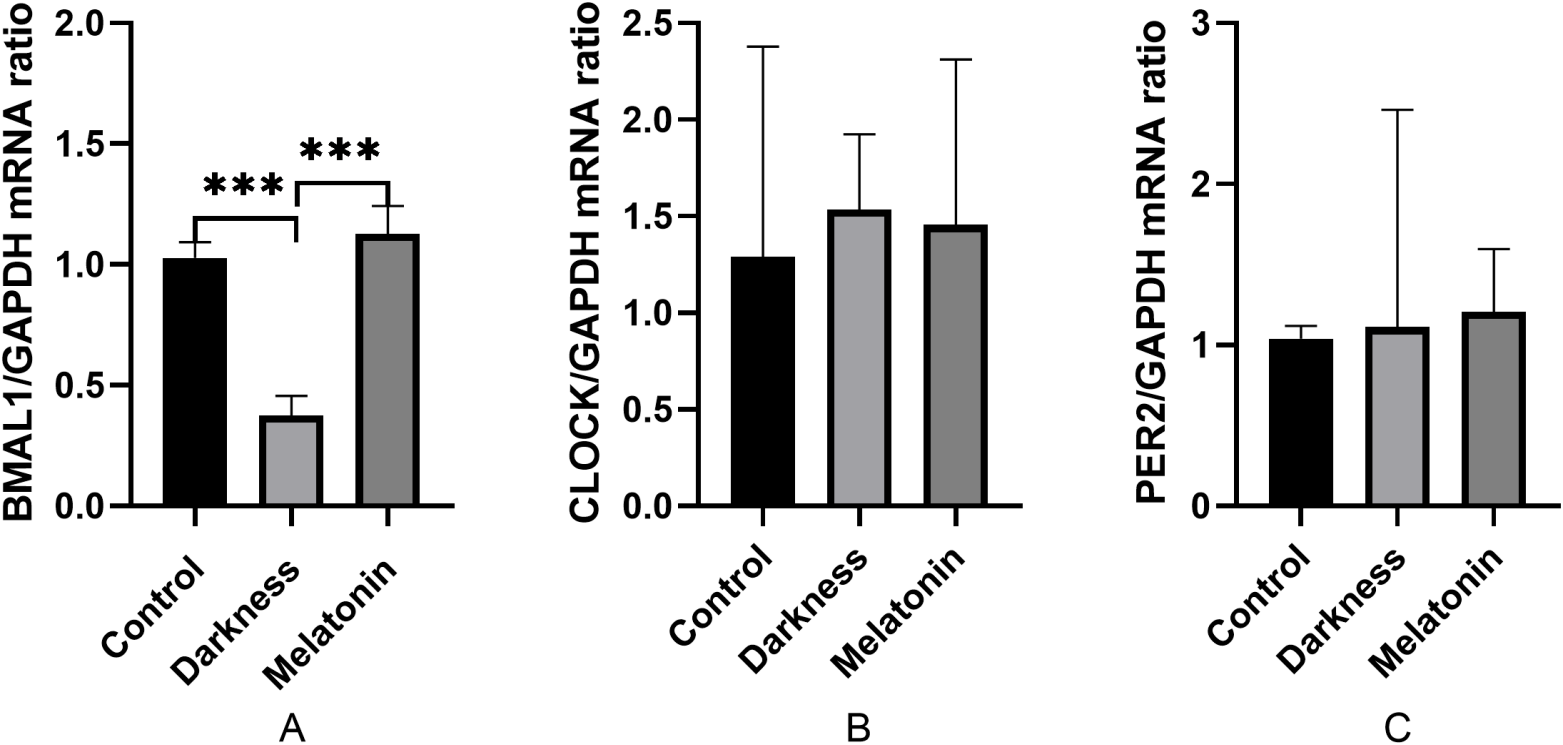
mRNA abundances of BMAL1 **(A)**, CLOCK **(B)**, and PER2 **(C)** in rat PBMC detected by qPCR. GAPDH was used as a loading control for qPCR analyses. N=6 per group. Statistical analysis was performed with one-way ANOVA followed by Newman–Keuls multiple comparison test. Data are expressed as mean± SD from 3 experiments. ***P < 0.001.

As illustrated in **Figure 8**, the mRNA expression of BMAL1 decreased significantly in the darkness contrasting to the control group (*P* < 0.05). Notably, Melatonin administration substantially reversed the mRNA expression of BMAL1 (*P* < 0.05). The mRNA expression of CLOCK and PER2 did not differ among the three groups (*P* < 0.05).

### Effect of melatonin on the mRNA expression of MTNR1A, MTNR1B, AR, and CYP19A1 in the ovarian tissues

**Figure 9:**
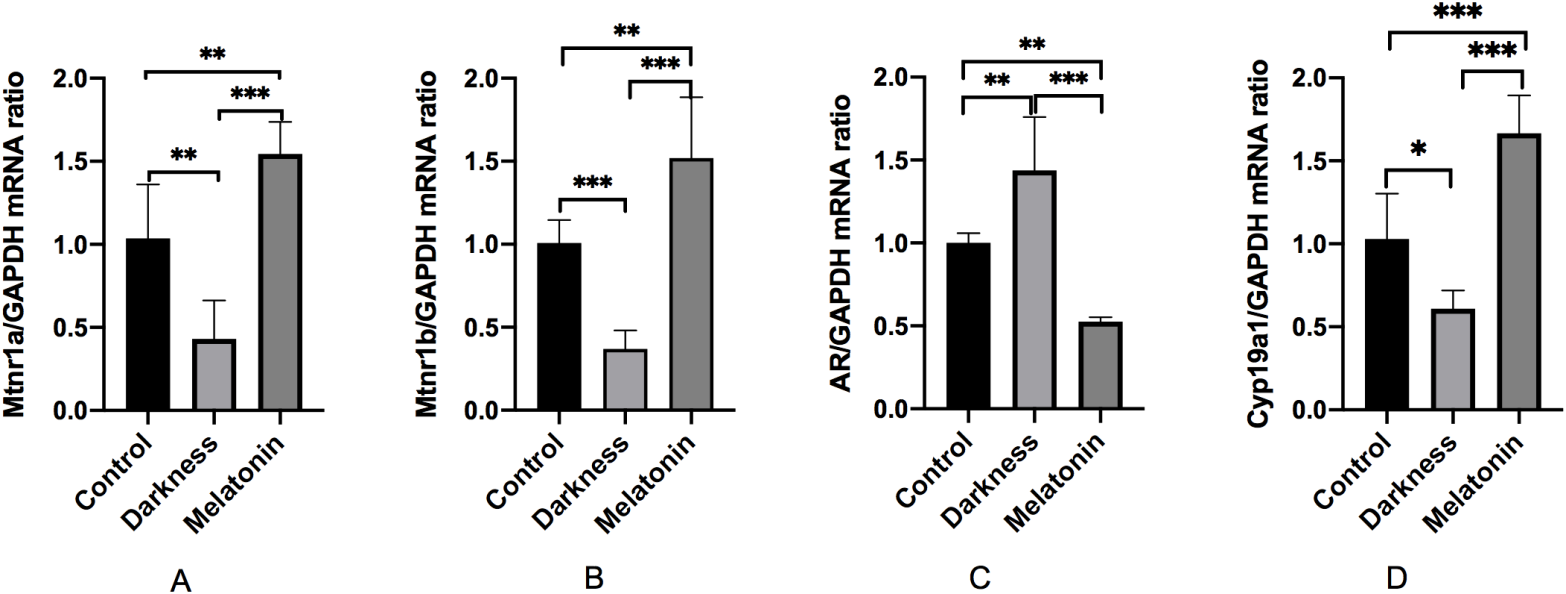
mRNA abundances of MTNR1A **(A)**, MTNR1B **(B)**, AR **(C)**, and CYP19A1 **(D)** in rat ovary tissues detected by qPCR. GAPDH was used as a loading control for qPCR analyses. N=6 per group. Statistical analysis was performed with one-way ANOVA followed by Newman–Keuls multiple comparison test. Data are expressed as mean± SD from 3 experiments. *P < 0.05, **P < 0.01, and ***P < 0.001.

As illustrated in **Figure 9**, the mRNA expression of MTNR1A, MTNR1B, and CYP19A1 decreased significantly in the darkness group compared to that in the control group (*P* < 0.05), whereas the mRNA expression of AR was markedly increased in the darkness group (*P* < 0.05). Compared to the darkness group, the mRNA expression of MTNR1A, MTNR1B, and CYP19A1 increased significantly in the melatonin group (*P* < 0.05), whereas the mRNA expression of AR was markedly reduced in the melatonin group (*P* < 0.05).

## Discussion

The complex multifactorial etiology of PCOS presents challenges for the formulation of individualized therapeutic approaches. There is a persistent necessity for therapeutic interventions that address the underlying causes and yield efficacious outcomes. Therapeutic strategies targeting hyperandrogenism, a hallmark of PCOS, have been extensively explored. Prolonged darkness leads to hyperandrogenism in PCOS-like rat models (26). One relevant mechanism is related to the downregulation of MTNR1A, which disturbs AR expression and downstream CYP19A1 transcription (26). Studies have also revealed that dysregulation of the expression of some essential clock genes, such as BMAL1, is closely associated with hyperandrogenism (46). CYP19A1 has also been identified as an immediate objective of BMAL1 in PBMCs, and low expression of BMAL1 increases the activity of 5a-reductase, exacerbating hyperandrogenism (13). Recently, promising therapeutic interventions targeting circadian rhythm modifications to mitigate hyperandrogenism have been proposed. Similar to a rhythm synchronizer, melatonin may directly influence both peripheral and central clock characteristics. (12, 47). Clinical investigations have confirmed that melatonin supplementation can enhance sleep quality and significantly alleviate hyperandrogenism in women with PCOS (21, 22). However, the exact mechanism by which melatonin functions remain unclear.

First, we validated the anti-androgen effects of melatonin in a circadian dysrhythmia-induced PCOS-like rat model. PCOS-like rats were induced by long-term environmental exposure to darkness, as previously described (26). We observed high serum levels of total testosterone, estrous cycle irregularity, and polycystic ovarian malformations in the darkness group. These findings indicate that continuous darkness, an unusual condition causing circadian rhythm disruption, leads to changes resembling PCOS morbidity. After 8-week treatment, we found that melatonin reduced serum testosterone levels, restored the estrous cycle, and relieved abnormal ovary morphology.

To explore the underlying mechanism, we conducted network pharmacology analysis. Network pharmacology findings elucidated that melatonin could potentially ameliorate PCOS through multiple pathways and cellular processes. Fundamental biological mechanisms, such as the steroid hormone receptor Signaling pathway within cells, cellular response to estrogen stimulus, steroid catabolic processes, and intracellular ER Signaling pathways, demonstrate the diverse modulatory effects of melatonin on hormone production within cells. Based on the KEGG pathway enrichment analysis, it can be postulated that melatonin may exert beneficial effects by modulating circadian rhythm, reproductive processes, metabolic functions, and oocyte maturation. Perturbations in circadian rhythm can result in reproductive and metabolic disorders. Furthermore, research has demonstrated that melatonin possesses potent antioxidant and anti-inflammatory properties (48). Accordingly, we speculated that melatonin could alleviate reproductive disorders in PCOS by exerting multifaceted effects: melatonin, acting as an essential rhythm synchronizer, could lessen the reproductive and metabolically related symptoms of PCOS. Its strong antioxidant effects also protect oocytes from oxidative damage.

To determine the impact of melatonin on the circadian rhythm of rats, we detected the mRNA expression of some core clock genes in PBMCs. The mRNA expression of BMAL1 was markedly reduced in PBMCs in the dark group, implying that the irregular expression of circadian clock genes in PBMCs resembled that observed in individuals with PCOS (13). After 8-week treatment, we found that melatonin reversed BMAL1 expression. Moreover, we found that PCOS and melatonin share 37 targets. Among the 37 targets, MTNR1A, AR, and CYP19A1 were closely related to hyperandrogenism, as stated above. Molecular docking and MD also showed the highest affinity and stability between AR, CYP19A1 and melatonin, indicating that these targets may serve crucial functions in treating PCOS, but further experimental verification was necessary. Due to the fact that the main and core source of androgens in PCOS patients with hyperandrogenism is the ovaries, so we further employed experimental validation to measure the expression of these genes in the ovarian tissues. We observed downregulated expression of MTNR1A, MTNR1B, and CYP19A1, overexpression of AR in ovary tissues in the darkness group, which were also reversed by melatonin treatment. The results of our study imply that melatonin may relieve hyperandrogenism in darkness-induced PCOS by regulating MTNR1A, MTNR1B, AR, and CYP19A1 expression.

It is worth noting that the 10 mg/kg/day dose of melatonin, commonly used in pre-clinical rodent studies, is substantially higher than typical physiologic doses. In healthy humans, endogenous melatonin production by the pineal gland follows a day-night cycle: secretion begins around 9–10 PM in response to darkness and peaks between 2–4 AM, with serum concentrations (Cmax) typically ranging from 60–100 pg/mL (49). For long-term management of adult insomnia disorder, clinical trials have used oral melatonin doses ranging from 0.3 to 8 mg/day (50). Pharmacokinetic studies indicate that exogenous melatonin administration in humans yields Cmax values ranging from 170 pg/mL to several thousand pg/mL (51). Notably, a 20 mg/day oral dose demonstrated potential neuroprotective benefits and improved sleep quality in breast cancer patients undergoing chemotherapy, whereas a 6 mg/day dose did not show similar cognitive benefits (52, 53). These findings suggest that super-physiological melatonin doses may exert multiple effects, such as circadian rhythm entrainment and anti-inflammatory actions. Considering rats exhibit ~3-fold faster melatonin clearance than humans, the 10 mg/kg/day dose ensures target engagement. Nevertheless, results from our animal experiments still should be interpreted with caution.

Nonetheless, this study had limitations. For instance, while the LH/FSH ratio provides more comprehensive information than LH level alone, we opted not to measure FSH or other laboratory parameters such as AMH. This decision was made primarily due to the study’s focus on verifying melatonin’s efficacy against hyperandrogenism, and also considering funding constraints. We will explore these indicators further in a follow-up study. Furthermore, a limitation of this study was the concurrent administration of melatonin with continuous darkness. Future studies will address this by distinguishing between prophylactic and therapeutic interventions. The pharmacological effects of melatonin cannot be fully anticipated, owing to the scarcity of information in publicly accessible databases. Moreover, it should be noted that the in silico analyses (such as molecular docking and structural prediction) in this study focused on the functional active sites of proteins, while the experimental validation only detected gene expression at the mRNA level. Since there may be differences in post-transcriptional regulation (such as translation efficiency, protein modification, or degradation) between mRNA abundance and protein activity, the direct correlation between the two should be interpreted with caution. This limitation suggests that future studies should combine protein activity assays (such as enzyme activity analysis and Western blot) or cell function experiments to further validate the correspondence between computational predictions and actual biological effects.

## Conclusion

Integrating network pharmacology, molecular docking, molecular dynamics simulations, and experimental validation, this study sheds light on potential mechanisms by which melatonin may ameliorate PCOS, especially hyperandrogenism. While these results suggest that melatonin’s therapeutic effects in PCOS involve the mitigation of androgen excess, further studies are warranted to validate these targets in human pathophysiology and explore their clinical translatability. Collectively, this work provides a promising foundation for understanding melatonin’s actions in circadian dysregulation-associated PCOS and highlights novel targets for future mechanistic and therapeutic investigations.

## Data Availability

The original contributions presented in the study are included in the article/**Supplementary Material**, further inquiries can be directed to the corresponding authors.
