# Supplementary figures and images for: Exploration of the molecular mechanism of melatonin against polycystic ovary syndrome based on a network pharmacology approach and experimental validation
Source: Front Endocrinol (Lausanne). 2025 Aug 5;16:1528518. doi: 10.3389/fendo.2025.1528518 (PMC12361247; doi:10.3389/fendo.2025.1528518)

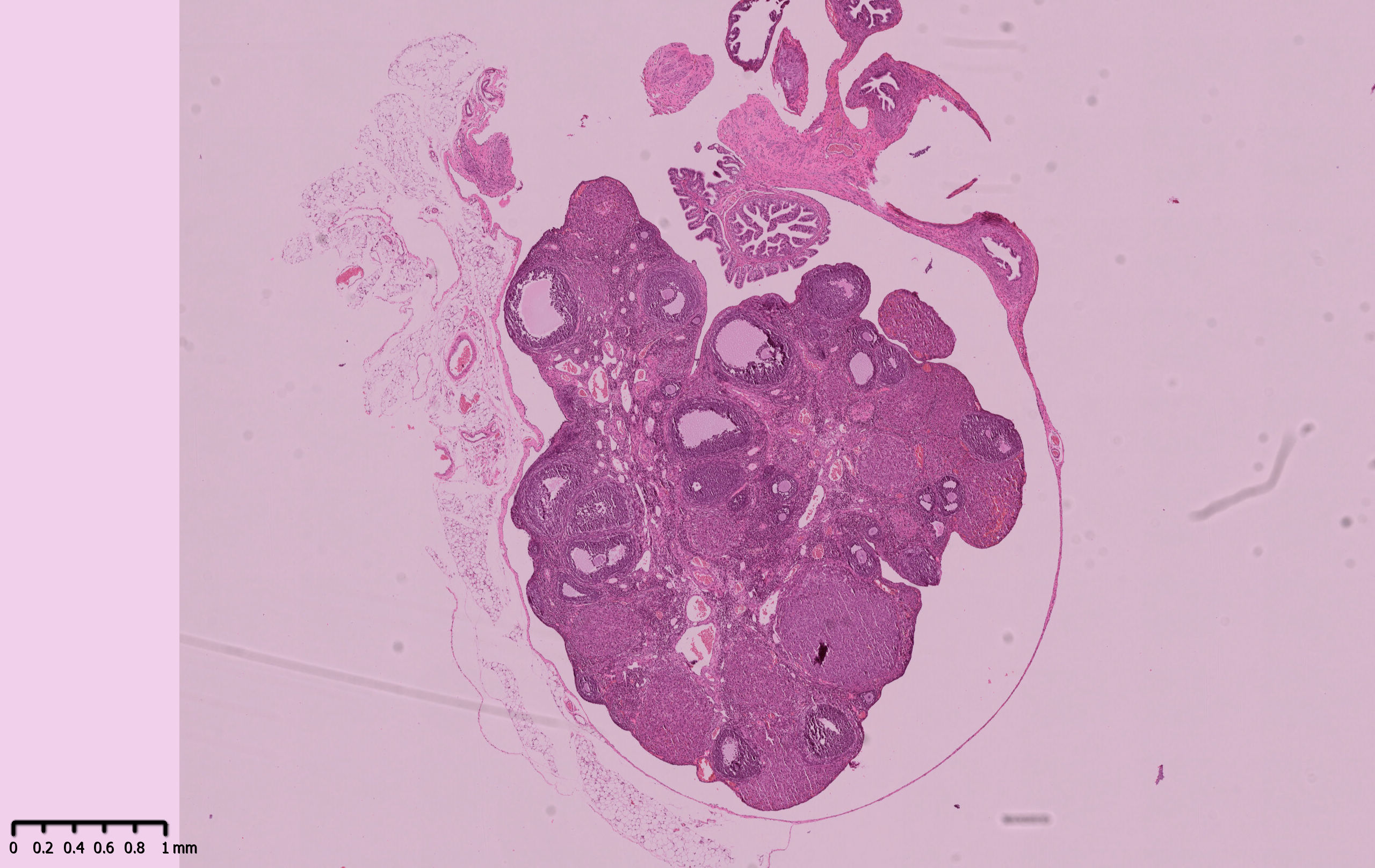

Supplement: Supplementary file 1 [file DataSheet1.zip › Raw data/HE/control1.jpg]

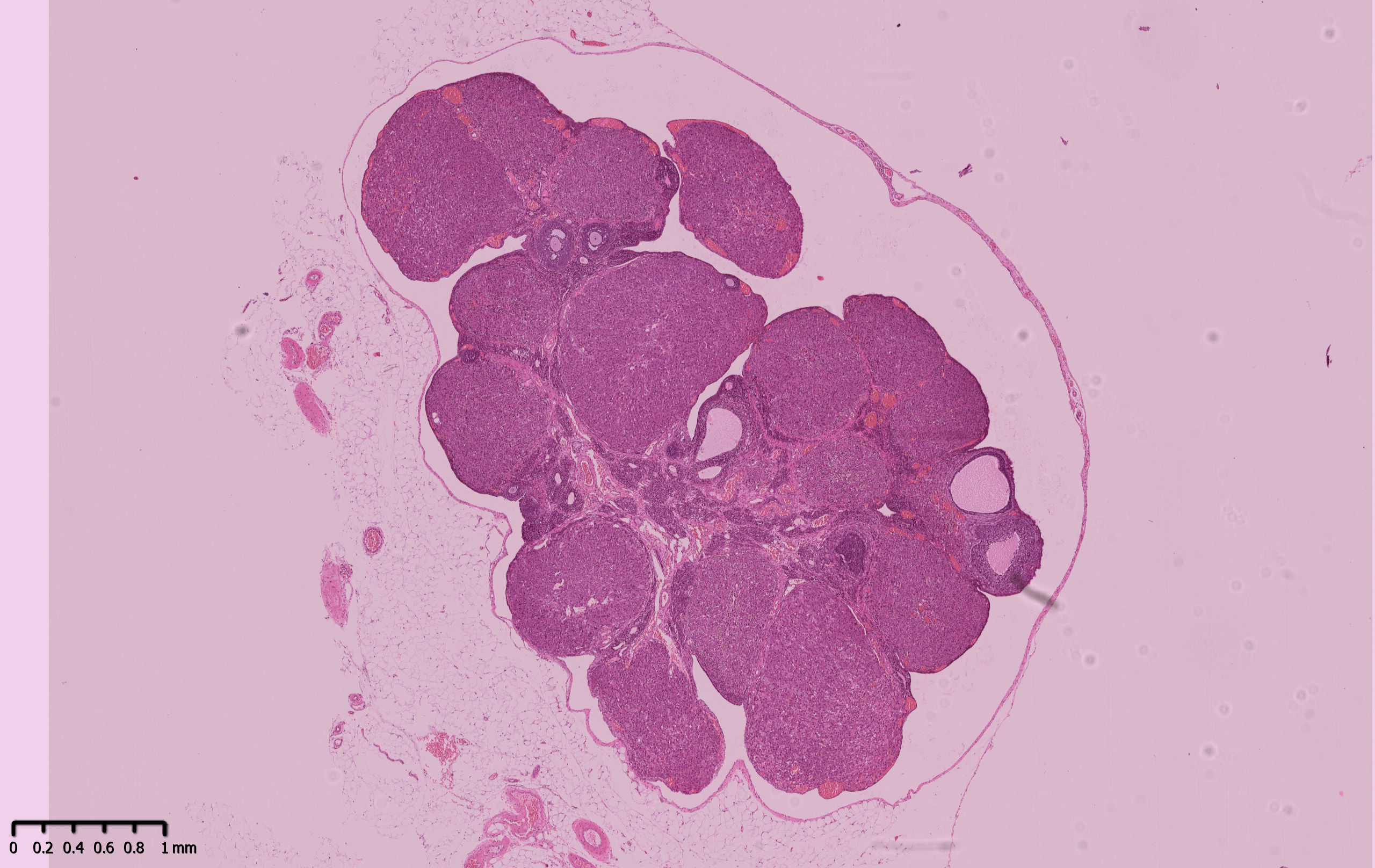

Supplement: Supplementary file 1 [file DataSheet1.zip › Raw data/HE/control2.jpg]

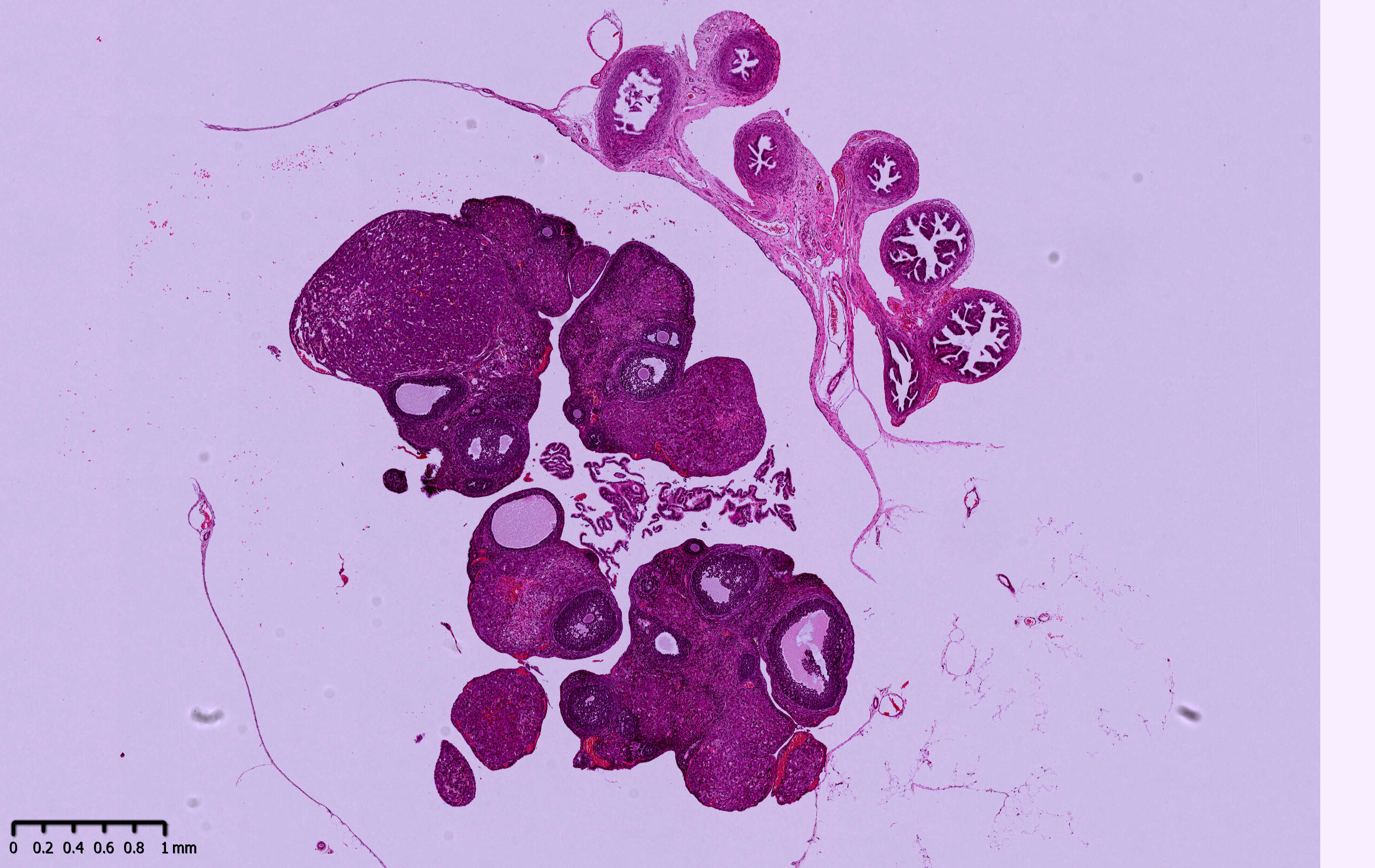

Supplement: Supplementary file 1 [file DataSheet1.zip › Raw data/HE/control3.jpg]

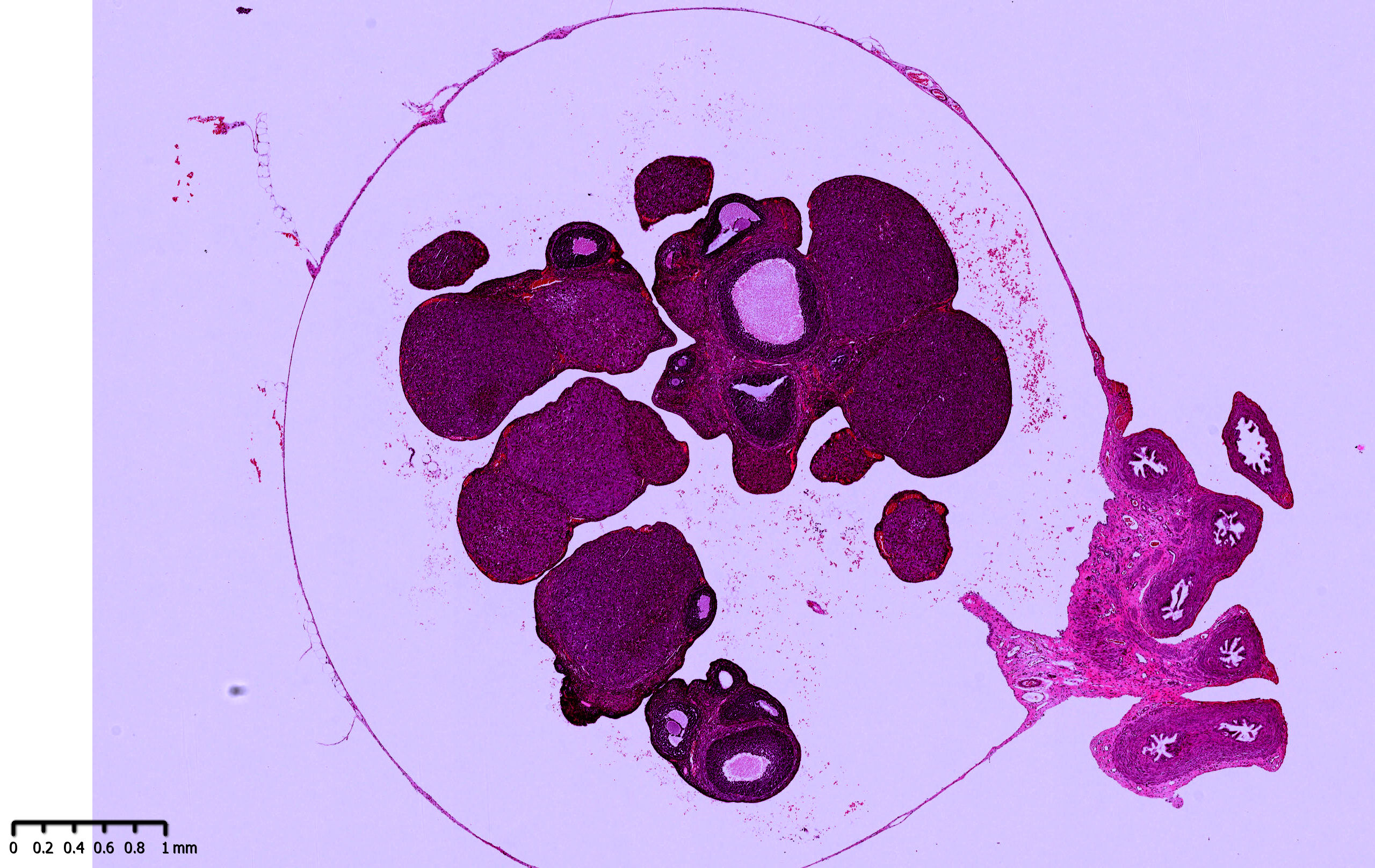

Supplement: Supplementary file 1 [file DataSheet1.zip › Raw data/HE/Darkness1.jpg]

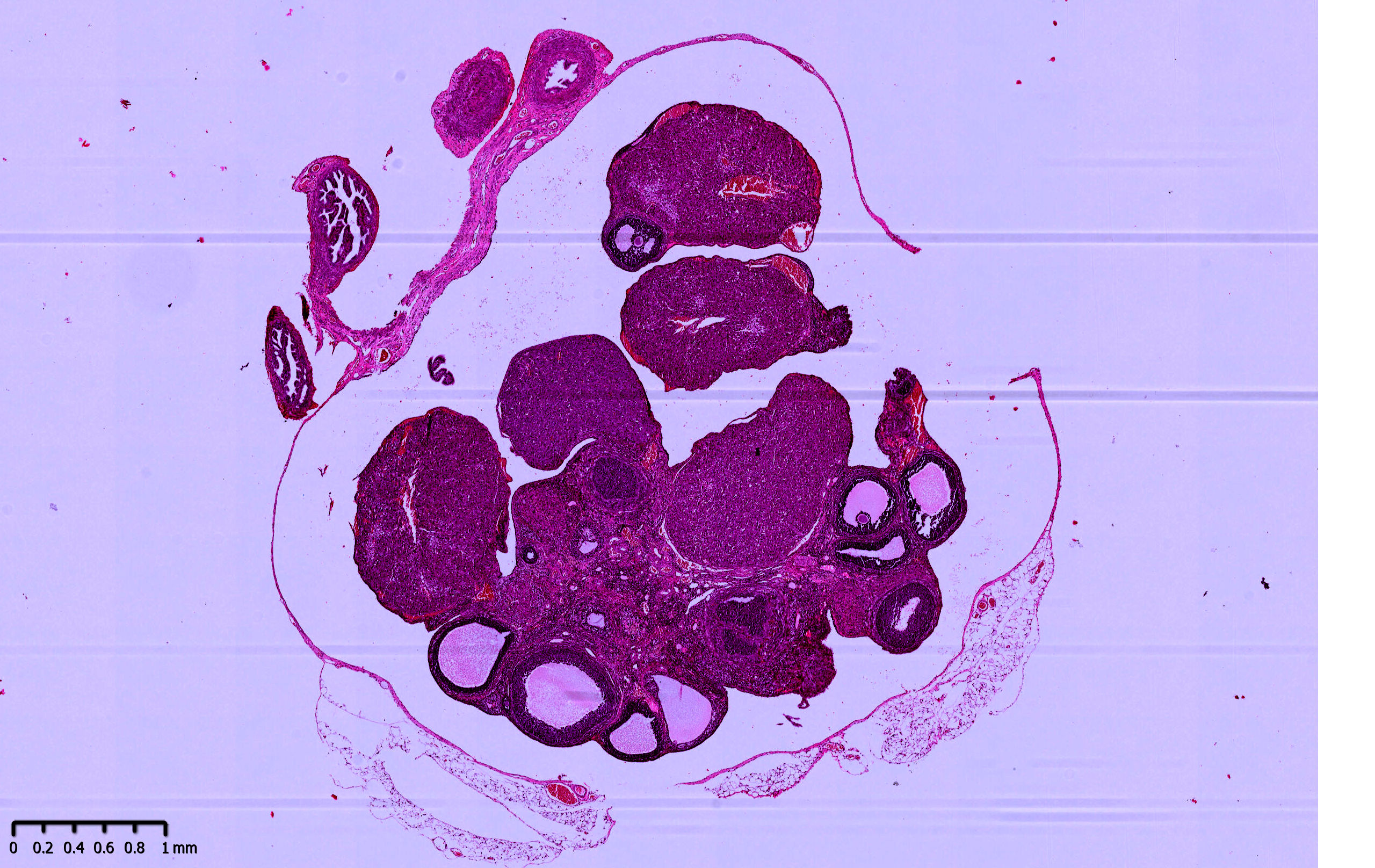

Supplement: Supplementary file 1 [file DataSheet1.zip › Raw data/HE/Darkness2.jpg]

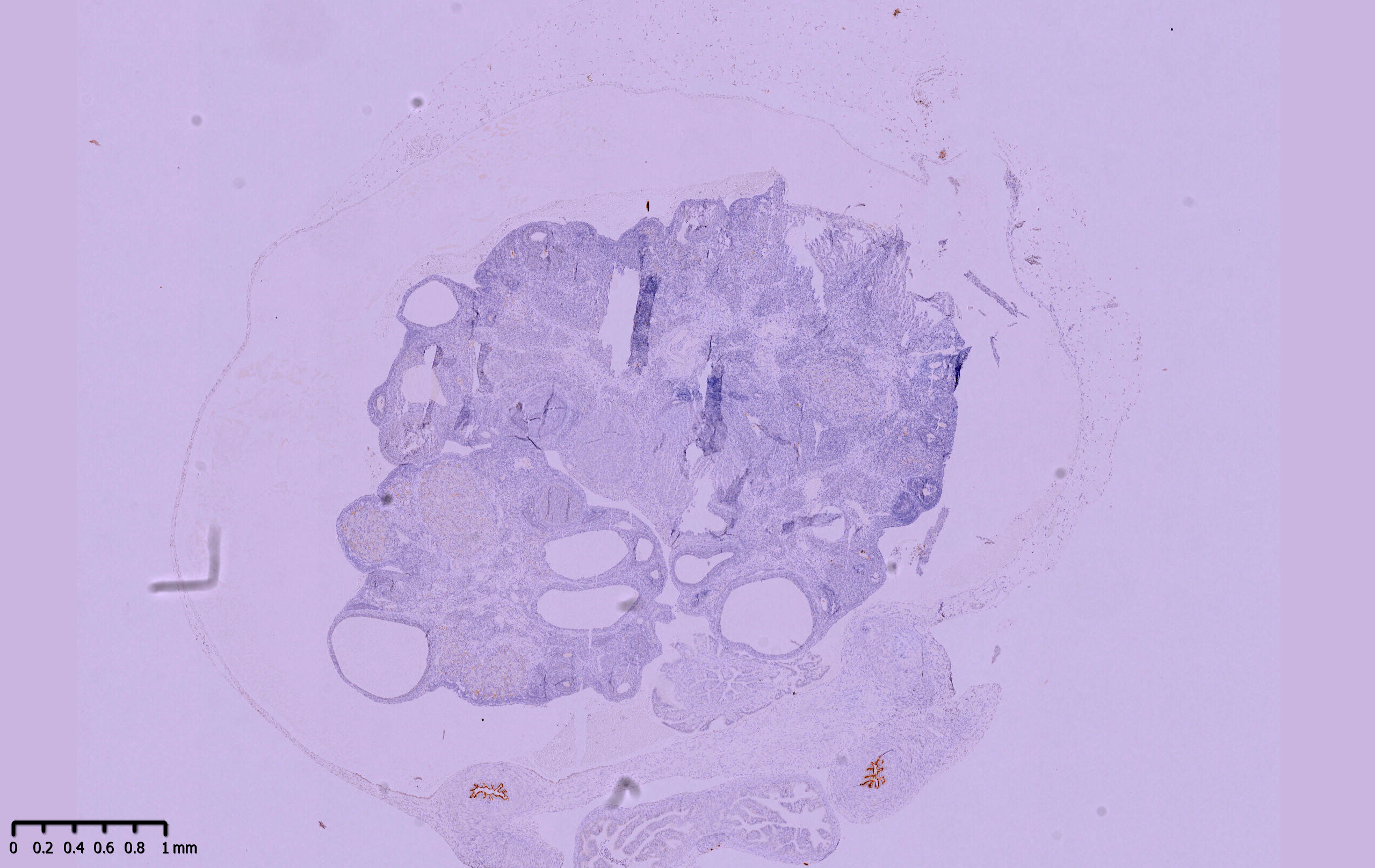

Supplement: Supplementary file 1 [file DataSheet1.zip › Raw data/HE/Darnness3.jpg]

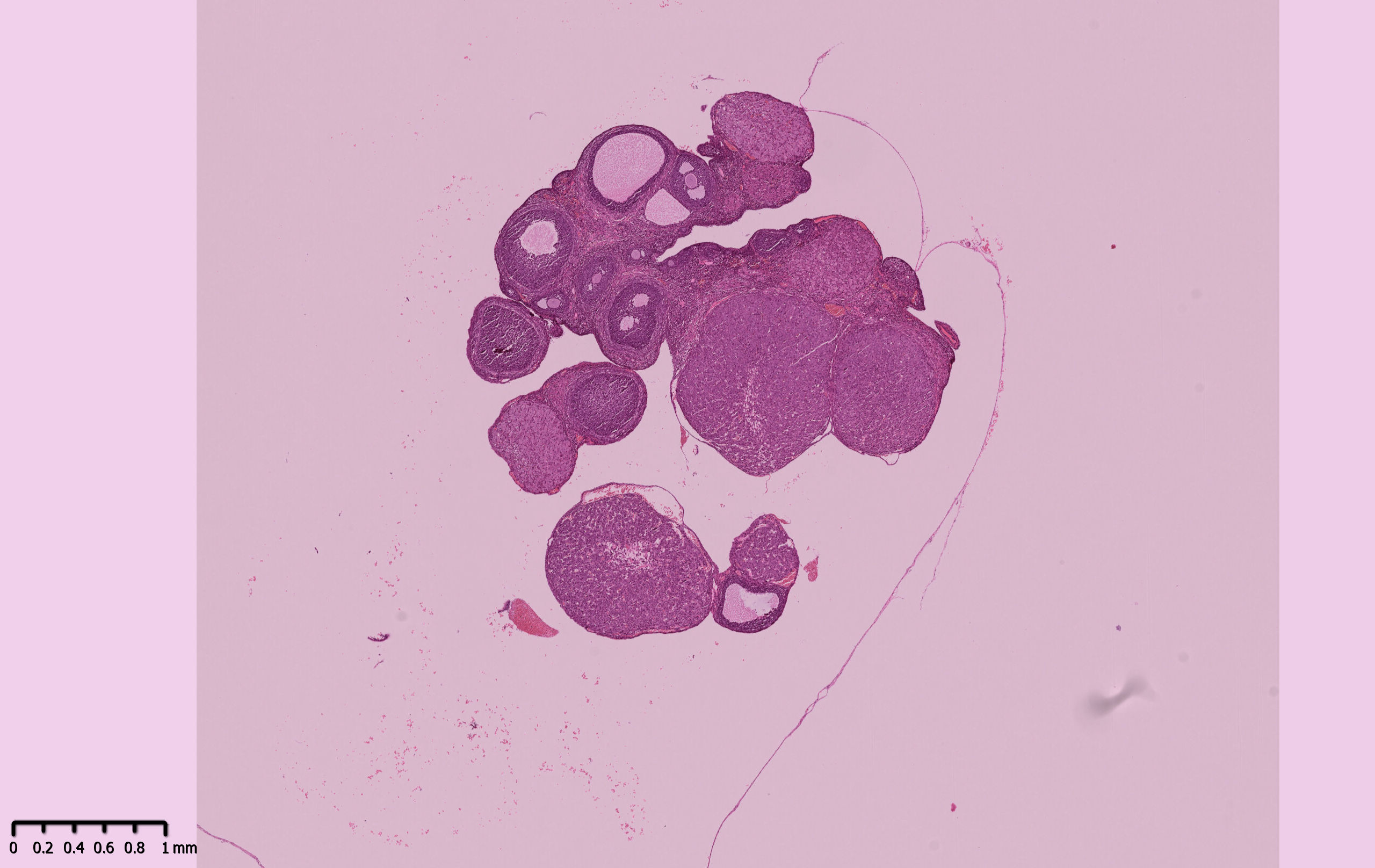

Supplement: Supplementary file 1 [file DataSheet1.zip › Raw data/HE/melatonin1.jpg]

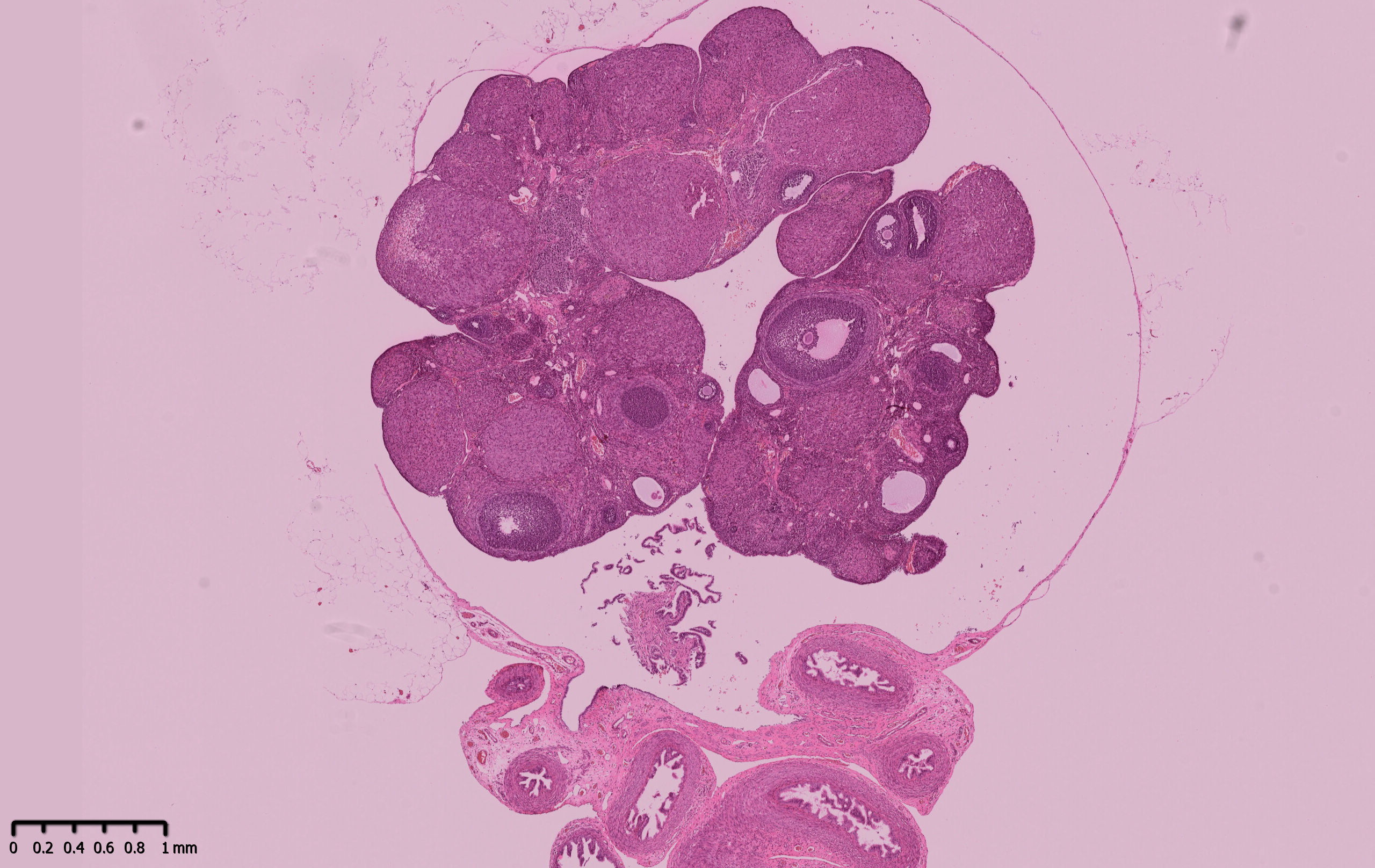

Supplement: Supplementary file 1 [file DataSheet1.zip › Raw data/HE/melatonin2.jpg]

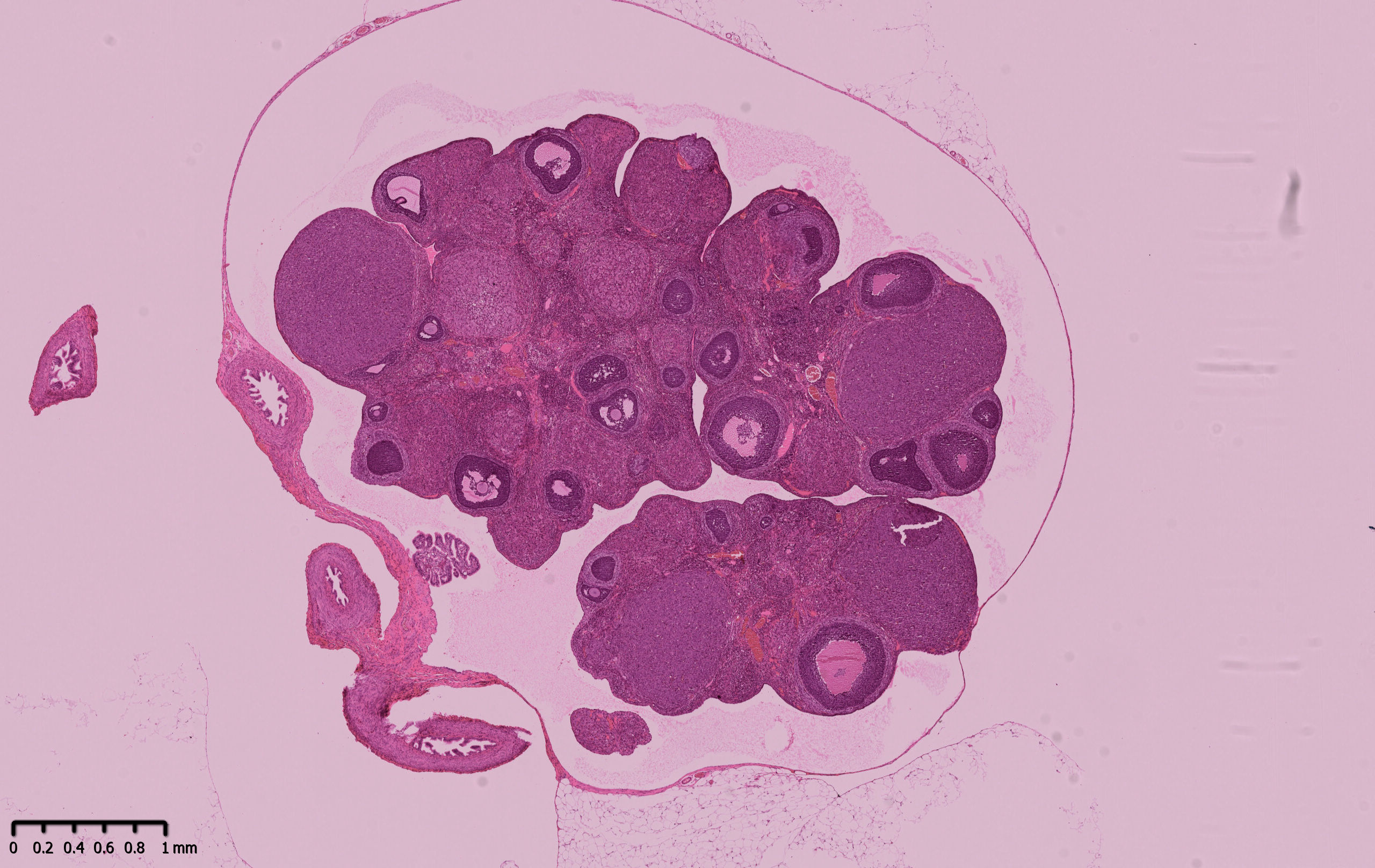

Supplement: Supplementary file 1 [file DataSheet1.zip › Raw data/HE/melatonin3.jpg]

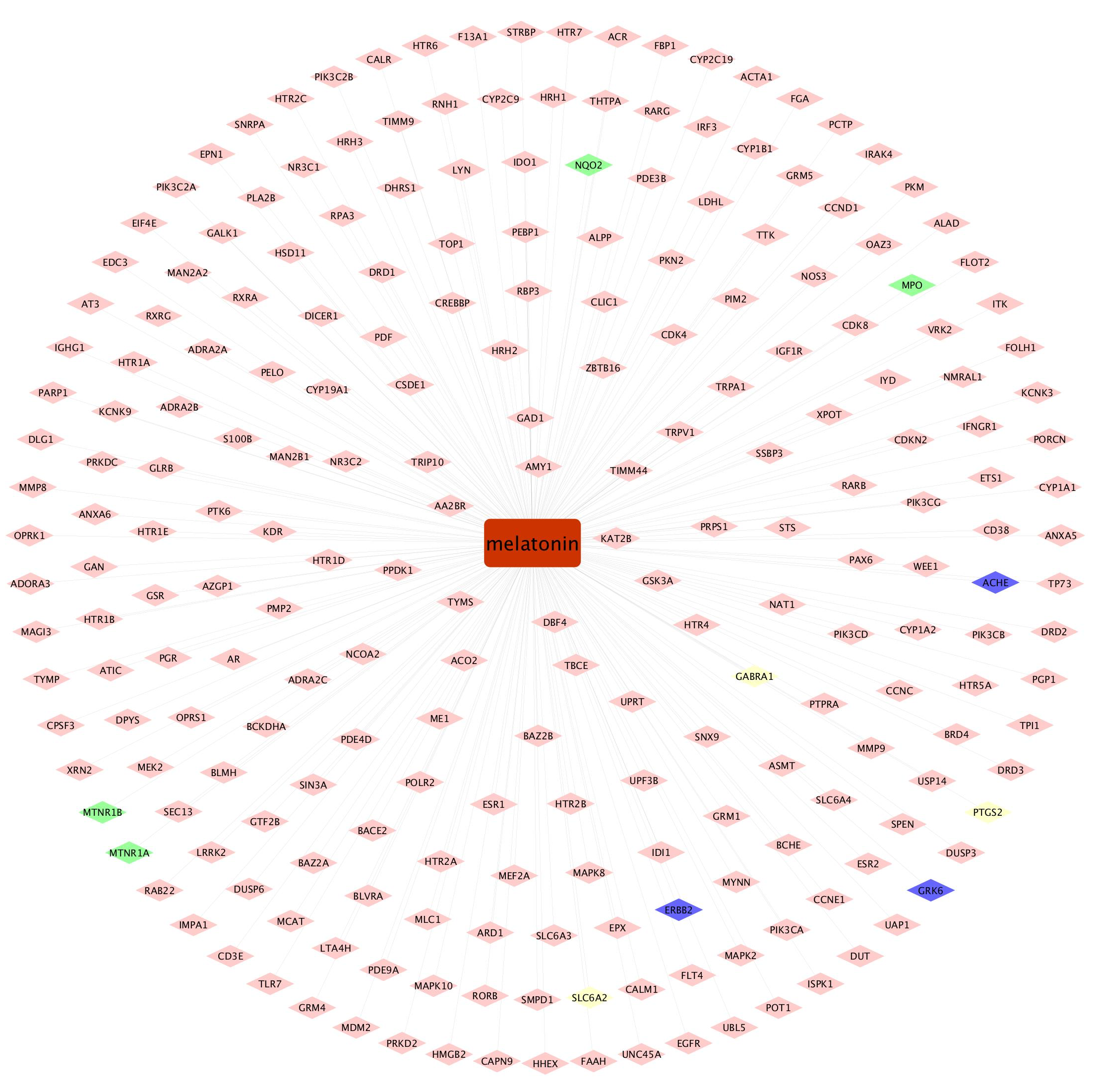

Supplement: Supplementary file 3 [file Image1.tiff]

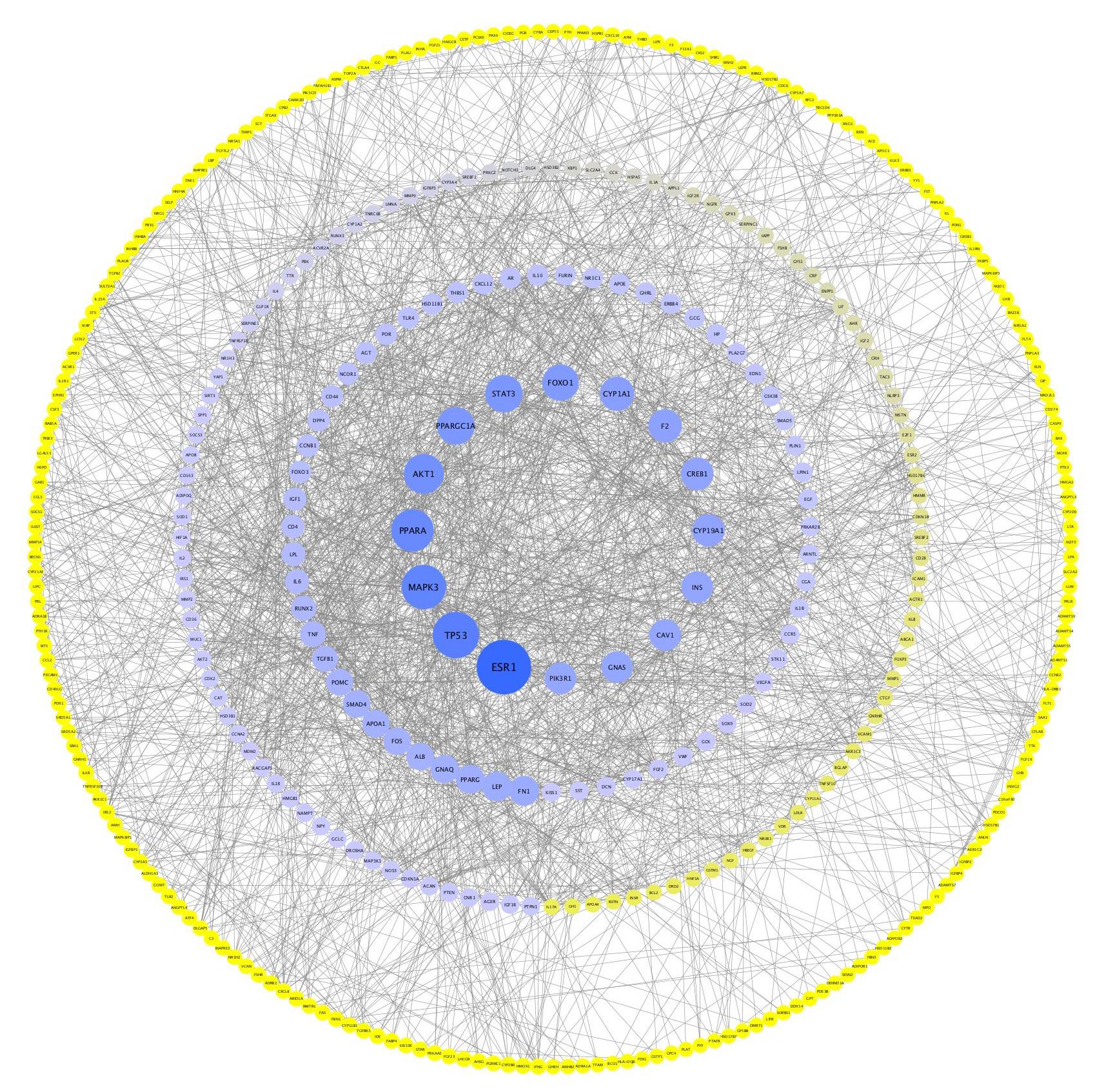

Supplement: Supplementary file 4 [file Image2.tiff]
