# Supplementary material for: Exploration of the molecular mechanism of melatonin against polycystic ovary syndrome based on a network pharmacology approach and experimental validation
Source: Front Endocrinol (Lausanne). 2025 Aug 5;16:1528518. doi: 10.3389/fendo.2025.1528518 (PMC12361247; doi:10.3389/fendo.2025.1528518)
Supplement: Supplementary file 2 [file DataSheet2.pdf]

动物实验伦理审查合格证明

Certificate of Ethical and Welfare Review of Animal Experiments

批准编号 Approval No.	20220815-11	终审编号 Review No.	IACUC-202212-10

经过实验动物管理与伦理委会审核，该项目动物实验过程符合动物保护、动物福利和伦理原则，符合国家实验动物福利伦理的相关规定。

According to the audit of the laboratory animal management and ethics committee, the animal experiment process of this project conforms to the principles of the animal protection, the animal welfare and the ethics as well as the related stipulation on national experimental animal welfare ethics.

实验名称 Protocol Title	加味桂枝甘草龙骨牡蛎汤调控生物钟改善昼夜节律紊乱PCOS大鼠高雄激素血症的机制研究				
申请人姓名 Applicant	程冉	职称/学位 Title/Degree	博士	邮箱 Email	rindy_cr@163.com
课题负责人 Principal Investigator	程冉	职称/学位 Title/Degree	博士	邮箱 Email	rindy_cr@163.com
院系（部门） Department	杭州市中医院				
实验时间 Experimental period	2022-9-20 - 2022-12-11	实验动物使用许可证 Number of Animal Use Permit	SYXK（浙）2021-0012		
动物实验研究中心主治兽医 LARC Attending Veterinarian	[signature]			日期 Date	2022.12.12
动物实验研究中心IACUC主席 LARC IACUC Chairman	吕建敏			日期 Date	2022.12.12

浙江中医药大学实验动物管理与伦理委员会

Animal Ethical and Welfare Committee of ZCMU

日期(Date)：2022.12.12
